# Machine learning based study for the classification of Type 2 diabetes mellitus subtypes

**DOI:** 10.1186/s13040-023-00340-2

**Published:** 2023-08-22

**Authors:** Nelson E. Ordoñez-Guillen, Jose Luis Gonzalez-Compean, Ivan Lopez-Arevalo, Miguel Contreras-Murillo, Edwin Aldana-Bobadilla

**Affiliations:** 1 Cinvestav Tamaulipas, Carretera Victoria-Soto la Marina km 5.5, Victoria, 87130 Tamaulipas Mexico; 2 CONAHCYT-Centro de Investigación y de Estudios Avanzados del IPN, Unidad Tamaulipas, Carretera Victoria-Soto la Marina km 5.5, Victoria, Tamaulipas 87130 Mexico

**Keywords:** Diabetes, Diabetes subtypes, Data-driven, Classification

## Abstract

**Purpose:**

Data-driven diabetes research has increased its interest in exploring the heterogeneity of the disease, aiming to support in the development of more specific prognoses and treatments within the so-called *precision medicine*. Recently, one of these studies found five diabetes subgroups with varying risks of complications and treatment responses. Here, we tackle the development and assessment of different models for classifying Type 2 Diabetes (T2DM) subtypes through machine learning approaches, with the aim of providing a performance comparison and new insights on the matter.

**Methods:**

We developed a three-stage methodology starting with the preprocessing of public databases NHANES (USA) and ENSANUT (Mexico) to construct a dataset with *N* = 10,077 adult diabetes patient records. We used *N* = 2,768 records for training/validation of models and left the remaining (*N* = 7,309) for testing. In the second stage, groups of observations –each one representing a T2DM subtype– were identified. We tested different clustering techniques and strategies and validated them by using internal and external clustering indices; obtaining two annotated datasets *Dset A* and *Dset B*. In the third stage, we developed different classification models assaying four algorithms, seven input-data schemes, and two validation settings on each annotated dataset. We also tested the obtained models using a majority-vote approach for classifying unseen patient records in the hold-out dataset.

**Results:**

From the independently obtained bootstrap validation for *Dset A* and *Dset B*, mean accuracies across all seven data schemes were $85.3\%$ ($\pm 9.2\%$) and $97.1\%$ ($\pm 3.4\%$), respectively. Best accuracies were $98.8\%$ and $98.9\%$. Both validation setting results were consistent. For the hold-out dataset, results were consonant with most of those obtained in the literature in terms of class proportions.

**Conclusion:**

The development of machine learning systems for the classification of diabetes subtypes constitutes an important task to support physicians for fast and timely decision-making. We expect to deploy this methodology in a data analysis platform to conduct studies for identifying T2DM subtypes in patient records from hospitals.

**Supplementary Information:**

The online version contains supplementary material available at 10.1186/s13040-023-00340-2.

## Introduction

### Background

Usually, diabetes has broadly been categorized into *Gestational* (GDM), *Type 1* (T1DM), and *Type 2* (T2DM). GDM occurs during pregnancy and increases the chances of developing T2DM later in life. T1DM usually appear at early ages when the pancreas stops producing insulin due to an autoimmune response. The reasons why this occurs are still not very clear. It is very important to monitor the glucose levels of patients, as sudden changes might be life threatening. Patients with this type often need a daily dose of insulin to lower their blood glucose levels. T2DM is the most common type of diabetes encompassing 95% of diabetic patients, which are commonly adults with a sedentary lifestyle and poor quality diet. Despite it can be easily controlled in early stages, comorbidities might appear years later. Stages of T2DM are related to parameters such as glucose concentration, insulin sensitivity, insulin secretion, overweight, and aging. However, recent studies have found that not all patients present the same manifestations.

According to the International Diabetes Federation (IDF) [1], diagnostic guidelines for diabetes include two measures obtained from blood tests: glycated hemoglobin test (HbA$_\text {1C}$) and plasma glucose (PG) test. The latter can be obtained in three different manners: in fasting state called *Fasting Plasma Glucose* (FPG), from an oral glucose tolerance test (OGTT), which consists of administering an oral dose of glucose and measuring PG after two hours; and from a sample taken at random time (normally carried out when symptoms are present), called *Random Plasma Glucose* (RPG). A positive diagnosis is reached when either one of the following conditions holds (IDF recommends two conditions in absence of symptoms): (1) FPG $\ge$ 7.0 mmol/L (126 mg/dL), (2) PG after OGTT $\ge$ 11.1 mmol/L (200 mg/dL), (3) HbA$_\text {1C}$ $\ge$ 6.5%, or (4) RPG $\ge$ 11.1 mmol/L (200 mg/dL). These parameters allow to readily identify diabetic patients and, when combined with risk factors such as demographic, family history, dietary, etc. may help to predict the tendency of developing the disease or its related complications. Understanding the relation of distinct parameters to the pathology of the disease also helps scientists to develop new ways to treat it. In this regard, data-driven analysis provide powerful means to discover such relations.

With the relatively recent advent of big data supporting precision medicine [2], the understanding of diabetes changed from the classical division of T1DM,T2DM, and other minority subtypes, to the notion of a highly heterogeneous disease [3]. The field of research has directed the efforts towards the exploitation of available big data analysis – particularly from electronic health records – searching for refined classification schemes of diabetes [4]. Indeed, recent diabetes research has stressed the importance of underlying etiological processes associated to development of important adverse outcomes of the disease along with response to treatment [5–7]. Exploring the disease heterogeneity, a recent data-driven unsupervised analysis [8] found that T2DM might have different manifestations including five subtypes that were related to varying risks of developing typical diabetes complications such as kidney disease, retinopathy, and neuropathy. Based on Ahlqvist et al. data-driven analysis [8], in this paper we tackle the development of methods for classifying T2DM subtypes through machine learning approaches with the aim of providing a comparison and new insights on the matter. In short, the goals of the present study were:

- Construct a dataset using publicly available databases comprising a majority of mexican and other hispanic population.
- Obtain a characterized dataset with different T2DM subgroups by means of clustering algorithms and evaluate different clustering strategies through clustering validation indices.
- Train and validate classification models using different algorithms and data schemes.
- Test developed models with a hold-out dataset.

Compared to previous work, our study introduces the following contributions and main results:

- The development of classification models for T2DM subgroups. To our best knowledge, there is only one preceding study that tackled this issue [9].
- Validation of T2DM subtypes in a relatively large dataset predominantly composed of mexican and other hispanic population.
- An evaluation of clustering algorithms and strategies including indices to measure clustering quality.
- An assessment of performance of classification models for T2DM subtypes. This assessment included four algorithms, seven data schemes, two datasets, and two validation methods.
- Our models reached accuracies of up to 98.8% and 98.9% on both datasets. Simpler and faster algorithms such as SVM and MLP performed better. Models adjusted notably better to *Dset B* data and performance was more consistent within the schemes on this dataset. Both validation settings, bootstrap and 10-fold cross validation, yielded similar results.
- Finally, the simple majority vote implemented in the testing stage showed a great amount of consensus, providing class proportions akin to previously reported for other populations.

We will briefly review the subject of artificial intelligence works related to general diabetes and diabetes subgroup classification in the remaining of this Introduction section.

### Related work

Artificial intelligence – and particularly, machine learning – methods have been extensively applied within the biomedical field mainly for development of computational tools to aid in diagnosis of diabetes or its complications [10]. Data analysis has been applied in several diabetes studies, covering five different main fields: risk factors, diagnosis, pathology, progression, and management [11]. A number of studies deal with identification of diabetes biomarkers, generally by means of feature selection methods, such as evaluating filter/wrapper strategies [12], combining feature ranking with regression models to predict short-term subcutaneous glucose [13], and proposing new methods for feature extraction [14, 15] and generation [16]. Another subfield of research regarding machine learning applied to diabetes mellitus is devoted to detection/prediction of complications. With the rise of deep learning within the last decade, much of this work aims at predicting diabetic retinopathy through convolutional architectures and primarily analyzing retinal fundus images [17, 18], even deploying tools that are commercially available [19, 20]. Predictive tools for diabetic nephropathy were developed integrating genetic features with clinical parameters [21] and comparing performance of various models for detection of diabetic kidney disease [22]. Another major diabetic complications tackled with machine learning algorithms are cardiovascular disease [23], peripheral neuropathy [24], diabetic foot [25], and episodes of hypoglycemia [26, 27]. All of these classification/regression tasks are approached with varying machine learning methods, most of which are reviewed in [28, 29].

Until recently, diabetes mellitus was thought as a two-class disease, divided into the general Types I and II with some uncommon manifestations within them; such as monogenic types (e.g. Maturity Onset Diabetes of the Young - MODY, and neonatal diabetes) and secondary types (e.g. due to steroid use, cystic fibrosis, and hemochromatosis) [30]. As mentioned earlier, Ahlqvist et al. [8] introduced a novel subclassification of diabetes with a data-driven (clustering) approach. Using six variables (glutamate decarboxylase (GAD) antibodies, age at diabetes onset, body mass index, glycated hemoglobin, and homeostatic model assessment values for $\beta$ cell function and insulin resistance), they discovered five clusters (T2DM subtypes) that were dubbed as:

1. *Severe Autoimmune Diabetes* (**SAID**): It is probably the same as T1DM, but it is classified as a T2DM subtype, where the pancreas stops producing natural insulin by an autoimmune response. This is identified by the presence of GAD antibodies.
2. *Severe Insulin-Deficient Diabetes* (**SIDD**): It is similar to SAID, but the antibodies responsible for the autoimmune response are missing.
3. *Severe Insulin-Resistant Diabetes* (**SIRD**): the patients seem to produce a normal amount of insulin, but their body does not respond as expected, maintaining high blood sugar levels.
4. *Mild Obesity Related Diabetes* (**MORD**): It is related to a high body mass index, can be treated with a better diet and exercise when moderated.
5. *Mild Age Related Diabetes* (**MARD**): It is mostly present in elder patients, and corresponds to the natural body ageing.

For such subgroup identification, they used a cohort comprising 8,980 patients for initial clustering and then, found centroids were used to further cluster three more cohorts and replicate results. Importantly, these groups were associated with different disease progression and risk of developing particular complications.

Soon after this pioneer study, a number of works based on the proposed cluster analysis method emerged to replicate diabetes subgroup assessment within different cohorts (see Table 1). The subject was systematically reviewed in [31]. ADOPT and RECORD trial databases with international and multicenter clinical data comprising 4,351 and 4,447 observations, respectively, were analyzed in [32] to investigate glycaemic and renal progression. They found similar cluster results compared to those reported by Ahlqvist et al., but also that simpler models based on single clinical features were more descriptive to their same purposes. In a 5-year follow-up study of a german cohort with 1,105 patients [33], the authors evaluated prevalence of complications such as non-alcoholic fatty liver disease and diabetic neuropathy within diabetes subgroups after follow-up. Later, using this same german cohort, the authors assessed inflammatory pathways within the diabetes subgroups by analyzing pairwise differences in levels of 74 inflammation biomarkers [34]. In another study of the same german group [35], prevalence of erectile dysfunction among the five diabetes subgroups was researched. This complication presented a higher prevalence in SIRD and SIDD patients, suggesting that insulin resistance and deficiency play an important role in developing the dysfunction.

**Table 1:** Datasets and found proportions of diabetes subgroups reported in the literature

Reference	Database/study	Origin	*N*	SAID (%)	MARD (%)	MORD (%)	SIDD (%)	SIRD (%)
[8]	ANDIS	Sweden	8980	6.4	39.1	21.6	17.5	15.3
	SDR		1466	10.1	34.4	18.3	20.4	16.8
	ANDIU		844	7.6	41.7	21.0	14.6	15.2
	DIREVA (newly diagnosed)		878	9.9	47.3	22.8	8.9	11.2
	DIREVA (longer-term)		2607	14.7	41.0	19.8	14.0	10.6
[32]	ADOPT	International	4003	4.2	33.8	21.4	20.2	20.4
	RECORD		4148	NA	36.6	20.5	23.5	19.4
[33]	GDS	Germany	1105	22.4	34.9	29.2	2.5	11.0
[34]	GDS	Germany	414	21.0	35.0	32.0	3.0	9.0
[36]	Retrospective study	China	14624	6.2	30.9	21.6	24.8	16.6
[37]	Retrospective study	China	1152	4.4	21.4	34.6	20.5	19.0
[9]	NHANES	USA	1758	NA	^a^39.7	^a^21.4	^a^15.0	^a^23.9
	ENSANUT	Mexico	614	NA	15.8	32.2	41.9	10.1
	SIGMA	Mexico	1521	NA	16.8	34.3	41.1	7.8
	MSC	Mexico	331	NA	13.6	45.0	5.7	35.6
	CAIPaDi	Mexico	1608	NA	11.5	39.8	43.0	5.7
[38]	DCS	Netherlands	2953	NA	^b^48.1	17.6	12.7	21.6
	GoDARTS	Scotland	5509	NA	^b^45.7	19.3	17.3	17.7
	ANDIS	Sweden	7478	NA	^b^51.5	23.2	15.9	9.4
[39]	NHANES	USA	5489	NA	25.6	30.1	23.5	20.8
[35]	GDS	Germany	351	23.0	41.0	25.0	4.0	7.0
[40]	ORIGIN - All	International	7017	3.4	38.1	22.7	22.7	13.0
	ORIGIN - European	Europe	3361	3.3	40.9	22.3	17.6	15.8
	ORIGIN - Latin American	Latin America	2428	3.8	33.4	24.0	27.7	11.0
[41]	FDEMC	Japan	586	10.2	35.7	25.4	15.4	13.3

^a^ These percentages were recalculated merging results of NHANES cycles III and 1998-2004

^b^ These percentages correspond to the sum of MDH and MD groups as explained by the authors

DB abbreviations. *ANDIS* all new diabetics in scania, *SDR* scania diabetes registry, *ANDIU* all new diabetics in Uppsala, *DIREVA* diabetes registry Vaasa, *ADOPT* a diabetes outcome progression trial, *RECORD* rosiglitazone evaluated for cardiac outcomes and regulation of glycaemia in diabetes, *GDS* german diabetes study, *NHANES* national health and nutrition examination survey, *ENSANUT* encuesta nacional de salud y nutrición, *MSC* metabolic syndrome cohort, *DCS* diabetes care system, *GoDARTS* genetics of diabetes audit and research tayside study, *ORIGIN* outcome reduction with initial glargine intervention, *FDEMC* Fukushima diabetes, endocrinology, and metabolism cohort. *SAID* severe autoimmune diabetes, *MARD* mild age related diabetes, *MORD* mild obesity related diabetes, *SIDD* severe insulin-deficient diabetes, *SIRD* severe insulin-resistant diabetes

A couple of studies were carried out to validate the data-driven approach for diabetes subgroups in chinese population [36, 37]. The former consisted in a multicenter national survey with cross-sectional data comprising 14,624 records. These data showed similar distributions than those found by Ahlqvist et al. with a higher prevalence of SIDD class. The latter recruited 1,152 inpatients of a tertiary care hospital. After performing clustering on the data, the proportions were similar for SIDD and SIRD, but in this case MORD assembled the majority of records, instead of MARD. A team of researchers [9] verified the reproducibility of diabetes subgroups by introducing classification models with trained Self-Normalized Neural Networks (SNNN). They clustered NHANES data to obtained a labeled dataset on which four input data models were fitted. These models were used later to classify data from four different mexican cohorts to assess risk for complications, risk factors of incidence, and treatment response within subgroups. In a subsequent work [39], with the purpose of assessing prevalence of diabetes subtypes in different ethnic groups in US population, the research team applied their SNNN models to classify an extended NHANES dataset comprising cycles up to 2018.

A replication and cross-validation study was performed in [38], the authors used an alternative input data scheme replacing HOMA2 values – originally used for clustering – with C-peptide along with high density lipoprotein cholesterol. Five clusters were produced with the proposed scheme, three of them showing good matching with MORD, SIDD, and SIRD; whereas the combination of the remaining two showed good correspondence to MARD. Cross-validation among three different cohorts exhibited fair to good cluster correspondence. Pigeyre et al. [40] also replicated clustering results of the original Swedish cohort using data from an international trial named ORIGIN. In this cohort, they investigated differences in cardiovascular and renal outcomes within the subgroups, as well as the varied effect of glargine insulin therapy compared to standard care in hyperglycemia. Finally, the risk of developing sarcopenia was evaluated in a Japanese cohort previously characterized using cluster analysis [41]. Among diabetes subtypes, SAID and SIDD patients exhibited higher risk for the onset of this ailment.

## Methods

**Fig. 1:**
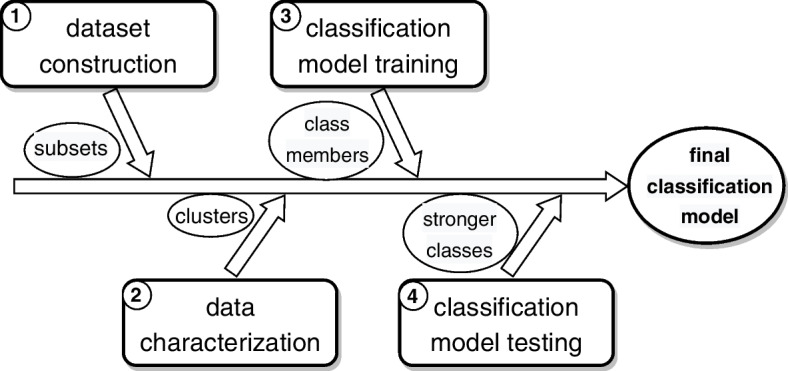
Overview of the general procedure applied in the study

Our interest was to explore different ways to obtain classification model variations for assigning T2DM subtypes to patients according to a set of attributes. This required us to characterize T2DM subtypes from existing databases, train these models and apply them to unseen patient records. The study followed a procedure with three main sequential stages shown in Fig. 1:

1. *Dataset construction*, where the tasks for acquiring, cleansing, merging and preprocessing the data are performed to get a tidy subset from databases. This subset is used for training, validating, and testing the clustering and classification models in the subsequent stage.
2. *Data characterization*, where diabetes patients (instances of the dataset) are segmented, yielding diabetes groups that are labeled according to the feature distribution patterns.
3. *Classification model training*, where different classification models are trained and validated using datasets from previous characterization; the obtained classification models are then used and evaluated by assigning T2DM subtypes to unseen patient records.

The best classification models were obtained according to different strategies varying co-related attributes. Next, in the following subsection, we describe these stages and steps more in detail.

### Dataset construction

The study was performed over real data (*NHANES* and *ENSANUT* databases). These data come from health surveys but was curated in several ways to obtain the better fitting of classification models.

- The *National Health and Nutrition Examination Survey* (NHANES) database [42], as its name suggests, is a U.S. national survey performed by the National Center for Health Statistics (NCHS), which in turn is part of the Centers for Disease Control and Prevention (CDC). It gathers information from interviews where people answer questionnaires covering demographic, nutritional, socioeconomic, and health related aspects. For some of the participants, physical examination and laboratory information are included. The database is divided in cycles, which after the NHANES III (1988 to 1998) are biennial. From NHANES can be obtained several datasets (views) for a vast number of works, depending on the interests of research. NHANES dataset that we assembled in the present work consists of the merging of cycles III (1988-1998) with all continuous NHANES cycles from 1999-2000 to 2017-2020. This latter cycle was the 2017-2018 cycle joined to the incomplete “pre-pandemic” cycle from 2019 to march 2020.
- The *Encuesta Nacional de Salud y Nutrición*^1^ (ENSANUT) [43], is the Mexican analogous of NHANES database. ENSANUT survey methodology, data gathering, and curation is carried out by the Center for Research on Evaluation and Surveys, which is part of the National Institute of Public Health (Mexican Ministry of Health). The database is the product of a systematic effort aiming to provide a trustworthy database to assess the status and tendencies of the population health condition, along with utilization and perception of health services. Starting in 1988 as the National Nutrition Survey, it was until 2000 that became a six-year survey (with some special issues) that included health information such as anthropometric measures, dietary habits, clinical history, vaccination, common diseases, and laboratory analysis (in some issues). Similarly to NHANES, several views can be obtained focusing on specific attributes. ENSANUT dataset that we have used here included the cycles 2006, 2016, and 2018.

From both databases we selected a subset of demographic, medical history, anthropometric, and laboratory variables (see Table 9). Importantly, C-peptide and Glucose2 were available in NHANES for only some cycles. C-peptide was only available in NHANES cycles III and 1999-2004, whereas Glucose2 was only available in NHANES cycles III, 2001-2002, and 2005-2016.

After merging the versions of each database, we obtained an initial raw dataset with *N* = 224,807 patients. From this, we selected only adult patients (Age $\ge$ 20 years, *N*=172,909). We then performed a data wrangling workflow including the following tasks, see Appendix B for a detailed description.

1. *Data cleansing* consisted in replacing some invalid values with zeroes to represent absent values.
2. An *imputation process* to assign values to missing and needed variable inputs to records that otherwise would be dismissed. When handling data, it is very likely that some values are missing for many circumstances, such as the participants of the survey did not answer the questions, then their answers could not be included in the dataset, or the laboratory samples could not be analysed. We imputed missing values by using the *Multivariate Feature Imputation* procedure, which infer absent values based on values available in other attributes. The considered variables were *Weight*, *Height*, *Waist*, *HbA1c*, *Glucose1*, *Glucose2*, *Insulin*, and *Age at Diabetes Onset* by taking the median value returned by four regression techniques (see Appendix B for details).
3. A *selection* step to maintain only those records that met the inclusion criteria: *a)* being a diagnosed patient, or *b)* having OGTT glucose $\ge$ 200 (mg/dL), or *c)* having HbA$_{1C}$ $\ge$ 6.5 (%). Extreme values, i.e. those values that were apart for more than five standard deviations from their mean, were removed on each attribute.
4. *Scaling*. Due to variations in the ranges of values of selected attributes, the computations are generally biased. Thus, a scaling on those values is required. We transformed the selected attributes by means of *min-max* normalization and *z-score* standardization.

**Fig. 2:**
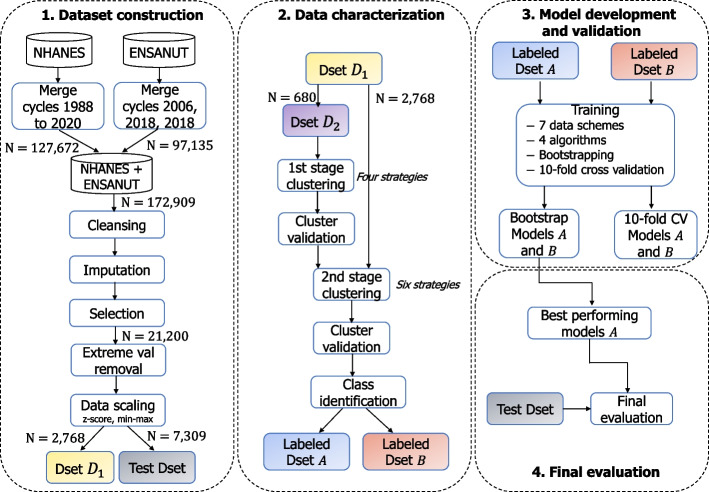
Main stages of the implemented procedure

As a result of the whole *dataset construction* process, a curated dataset was obtained combining NHANES and ENSANUT records. The process is illustrated on the left Panel in Fig. 2. The dataset was fully preprocessed according to the requirements of the study and, at this point, is ready for its utilization in data analysis algorithms. The final dataset comprised a total of 10,077 patient records that were split into a training/validation dataset termed $D_1$ (*N* = 2,768) and a hold-out dataset termed Test Dset (*N* = 7,309). $D_1$ consisted of the records including values for C-peptide variable, whereas *Test Dset* did not included these values.

### Data characterization

The objective of this stage was to characterize the selected instances in the curated dataset. The overall flow is depicted on Fig. 2 (central panel). Since this dataset was not labeled with any group or T2DM subtype, we applied clustering algorithms over selected attributes with the purpose of finding groups of instances in the dataset according to the similarities on values of the attributes. In a preliminary analysis, we explored three algorithms with different clustering approaches: partitional (*K-means* [44]), hierarchical (*agglomerative clustering* [45]), and density (*DBSCAN* [46]). Since these preliminary results (not included in this paper) demonstrated meaningful dissimilarities among DBSCAN and Agglomerative clusters with respect to those obtained with K-means, we determined to focus on the utilization of the latter.

Thus, we applied K-means to group T2DM patients into clusters, relying on the principle that similar patients in a cluster denote a T2DM subtype. We used a fixed number of groups (K = 4) corresponding to previously found diabetes subtypes [8], with the exception of SAID. We did not take this class into account considering all patients as being GADA negative. The five clinical features previously reported in the literature [8, 30] were taken into account. These features are: Age at Diabetes Onset (ADO), Body Mass Index (BMI), Glycated Haemoglobin (HbA$_{1C}$), and Homeostasis Model Assessment 2 [47] estimates of beta cell function and insulin resistance (HOMA2-%$\beta$ and HOMA2-IR, respectively). HOMA2 values are defined by computationally solving a system of empirical differential equations with software provided by the authors [48]. There are two types of HOMA2 values, one derived from FPG plus C-peptide, and the other derived from FPG plus Insulin. We used both types HOMA2 values, as will be explained later. Hereafter, we will refer to them as CP-HOMA2 and IN-HOMA2, respectively.

As mentioned earlier, dataset $D_1$ included only those records with C-peptide values (*N* = 2768) and thus, CP-HOMA2 measures can be computed for those records. Dataset $D_2$ (*N* = 680), in turn, consists of the subset of $D_1$ that only includes patients with less than five years of diabetes onset (i.e. AGE − ADO < 5). We carried out a two-stage clustering, first on $D_2$ and then, used the obtained centroids to cluster the remaining instances of $D_1$: those with five or more years of diabetes onset (i.e. the difference set $D_1-D_2$) in the second stage. In total, we tested four clustering strategies in the first stage (numbered 1.1 to 1.4) and six in the second stage (numbered 2.1 to 2.6). In both stages we aimed to contrast two overall clustering alternatives: (1) with centroid initialization or de novo clustering; and (2) taking each gender separately or both genders at once. In the first stage, we also tested the alternative of only assigning instances to initial centroids (i.e. no iteration) versus assigning and iterating until centroid convergence. Strategies 1.1 to 1.4 are thus defined as follows:

- **Centroid initialization using centroids provided by Ahlqvist et al.** [8]:
  (1.1) Only assigning instances to initial centroids.
  (1.2) Iterating until reaching centroid convergence.
- **De novo clustering with repeated K-means procedure:**
  (1.3) Each gender separately.
  (1.4) Both genders at once.

For 1.1 and 1.2 we took centroids reported by Ahlqvist et al. [8]. These centroids are defined by gender, therefore, centroid assignment is performed this manner in 1.1 and 1.2. De novo strategies 1.3 and 1.4 used a repeated K-means procedure, which consisted in several executions (51) of K-means. This procedure yielded a string with 51 positions, where each position holds one of {0, 1, 2, or 3} (the four groups). Hence, each string corresponds a group assignment pattern for each instance. Then, similarity among strings was compared to constitute the final four groups. This way, two identical strings mean that those instances were assigned the same groups across the 51 executions. Those strings not identical were grouped with their most similar instances. In all these executions, we used the K-means *scikit-learn* function with K = 4, 100 randomized centroid initializations (with *k-means++* function), and 300 maximum iterations.

After analyzing results from the first stage strategies, we selected strategies 1.2 and 1.4, according to intrinsic and extrinsic clustering validation indices (Appendix A). We then moved on to second stage clustering computing centroids from strategies 1.2 and 1.4. For both genders denoted by $\text {C}_{1.2}$ and $\text {C}_{1.4}$; and separated by gender (W)omen and (M)en denoted by $\text {C}_{1.2(W)}$, $\text {C}_{1.2(M)}$, $\text {C}_{1.4(W)}$, and $\text {C}_{1.4(M)}$. In second stage, we also carried out de novo clusterings with the repeated K-means procedure. Here, we included two forms of de novo clustering: in addition of using CP-HOMA2 parameters, we also tested a clustering using IN-HOMA2 parameters and scaling the data with *Min-Max* normalization, instead of *z-score*. Importantly, this latter strategy was the only one that implemented these changes. In this manner, the six strategies in second stage were:

- **Centroid initialization using centroids from first stage:**
  (2.1) With centroids $$\text {C}_{1.2}$$.
  (2.2) With centroids $\text {C}_{1.2(W)}$ and $\text {C}_{1.2(M)}$.
  (2.3) With centroids $$\text {C}_{1.4}$$.
  (2.4) With centroids $\text {C}_{1.4(W)}$ and $\text {C}_{1.4(M)}$.
- **De novo clustering with repeated K-means procedure:**
  (2.5) With CP-HOMA2 values.
  (2.6) With IN-HOMA2 values and *Min-Max* normalization.

Strategies 2.1 to 2.4 used centroids found in first stage for dataset $D_2$ and thus, only cluster the remaining instances in $D_1$. Strategies 2.5 and 2.6 cluster the whole dataset $D_1$ without taking into account previous results from first stage. Again, we evaluated the results by means of intrinsic and extrinsic validation indices selecting strategies 2.5 and 2.6 as the best performing ones. At the end of second stage clustering, we obtained two labeled datasets from $D_1$, named as *Dset A* and *Dset B*, from groups obtained from clustering 2.5 and 2.6, respectively. The matching of groups with labels of T2DM subtypes was performed by comparing the obtained attribute distribution patterns against those reported in the literature [8, 9, 30], as will be further explained in Results section.

### Model development and evaluation

Clustering in previous stage helped us to find out how patients can be grouped on T2DM subtypes; each patient was labeled according to its corresponding T2DM subtype. In this section, a subset of the dataset was used to train classification algorithms to learn how to identify unseen patients of the same dataset, not used for training. We developed classification models in two pathways, one for each annotated dataset (*Dset A* and *Dset B*; see upper-right panel in Fig. 2). On both pathways, we considered seven classification schemes, according to different selections of attributes in the input data. First, we used *bootstrapping* to validate models on both pathways, and then performed a second validation of best performing algorithms using *stratified 10-fold cross validation*. Four classification algorithms were explored: Support Vector Machine, K-Nearest Neighbors, Multilayer Perceptron, and Self-Normalized Neural Networks (see Appendix A for description). Finally, we used models obtained in the validation stage to classify subjects from the hold-out dataset.

#### Classification schemes

We explored how classification algorithms behave fed with different input data. The seven classification schemes denoted by S1 to S7 are the following:

- **S1.** ADO, BMI, HbA$_\text {1C}$, and CP-HOMA2-%$\beta$ and CP-HOMA2-IR.
- **S2.** ADO, BMI, HbA$_\text {1C}$, and IN-HOMA2-%$\beta$ and IN-HOMA2-IR.
- **S3.** ADO, BMI, FPG, and IN-HOMA2-%$\beta$ and IN-HOMA2-IR.
- **S4.** ADO, BMI, HbA$_\text {1C}$, FPG, and C-peptide
- **S5.** ADO, BMI, HbA$_\text {1C}$, FPG, and insulin
- **S6.** ADO, BMI, HbA$_\text {1C}$, HOMA-%$\beta$, and HOMA-IR.
- **S7.** ADO, BMI, HbA$_\text {1C}$, METS-IR, and METS-VF.

Note that all schemes include ADO and BMI and, with exception of scheme S3, all include also HbA$_\text {1C}$. Attributes that were interchanged within schemes are those related with pancreatic beta cell function and insulin resistance (i.e. HOMA measures and their related input variables: Glucose and C-peptide/insulin). Notice that schemes S1 and S2 consist of the same attributes on which *Dset A* and *Dset B* were respectively clustered. Scheme S3 is the same as S2 with HbA$_\text {1C}$ replaced by FPG. Schemes S4 and S5 substitute HOMA2 measures in schemes S1 and S2 with their respective input attributes. Scheme S6 makes use of a previous HOMA model [49] that uses simple formulas for approximating beta cells function and insulin resistance. Finally, scheme S7 applies *Metabolic Scores for Insulin Resistance* (METS-IR) [50] and *Visceral Fat* (METS-VF) [51], which are respectively proposed measures of insulin resistance and intra-abdominal fat content. Schemes S1, S2, S3, and S7 were implemented elsewhere [9] and here we added schemes S4, S5, and S6.

#### Training and validating models

In the validation stage, several models are trained to compare among them, obtain average metrics and choose the best ones. This task was carried out using two independent validation processes: *bootstrapping* and *stratified 10-fold cross validation*. The former is recommended for obtaining classification models that circumvent overfitting. This is a common undesired effect on classification models that occurs when the model memorizes the training dataset instead of learning to classify; therefore the statistics provided during training might not represent the actual performance of the model in real scenarios on unseen data. Bootstrapping helps to evaluate the model by randomly sampling a dataset with replacement to obtain the training data, and the rest of non sampled data, called *out-of-bag data*, to test its results. The process is repeated several times selecting different random samples each time. We chose to extract 1000 bootstrap samples and a distribution of metric values for each of the models.

Results obtained from bootstrapping validation were evaluated by means of classification metrics (see Appendix A) to select the best performing algorithm on each classification scheme. We then performed a stratified 10-fold cross validation process only on the selected algorithms. This process consists in randomly splitting the dataset in ten equitable partitions maintaining proportional number of records per each class. At each iteration of the cross validation process each partition was selected as the testing set and the remainder nine partitions combined as the training set. Unlike the bootstrapping procedure, where random sampling is processed at each iteration, in cross validation every model is validated on the exact same patient records, as the splitting is effected only at the beginning.

#### Final evaluation

After the validation stage, we saved the trained models of best performing algorithms in terms of accuracy, for each of the seven classification schemes. These were obtained from the bootstrapping procedure and thus, achieved the best accuracy among 1000 runs in each case. Since the hold out dataset (*N* = 7,309) did not contain C-peptide values, we classified it with five trained models from schemes S2, S3, S5, S6, and S7, which did not use this attribute. To obtain a final classification we applied the majority vote approach, breaking ties (i.e. two pair of schemes voting for two different classes each pair) by selecting the option of the model that achieved the highest accuracy during validation.

## Results

This section describes the results corresponding to the data characterization by following the different clustering strategies previously defined, the classification models obtained from the validation *Dset A* and *Dset B* using bootstrapping and cross-validation, and the final classification on the test dataset.

### Data characterization

For the first stage clustering, Table 2 shows the number of patients clustered on each group and the intrinsic validation values of the four clustering strategies applied on the dataset $D_2$. Overall, strategies 1.1, 1.2, and 1.4 obtained comparable scores and fairly similar distribution of patients among the groups, while clustering 1.3 produced considerably lower values on validation indices. As may be intuitively expected, allowing K-means to iterate until convergence after assigning initial centroids performed slightly better than the only-assign counterpart. In terms of these validation values obtained, performing a repeated K-means clustering without initial centroids and without gender separation outperformed the rest of strategies.

**Table 2:** Results for first stage clustering. Dataset $D_2$ (*N* = 680). *SIL* silhouette, *DB* Davies-Bouldin, *CH* Calinski-Harabasz. Best metric value achieved appears in bold

	Obs. per group				Intrinsic index		
Strat.	0	1	2	3	SIL	DB	CH
1.1	262	191	122	105	0.2013	1.5039	159.51
1.2	256	177	140	107	0.1951	1.4794	161.91
1.3	249	123	121	187	0.1545	2.0872	119.49
1.4	234	200	140	106	**0.2015**	**1.4554**	**167.34**

**Fig. 3:**
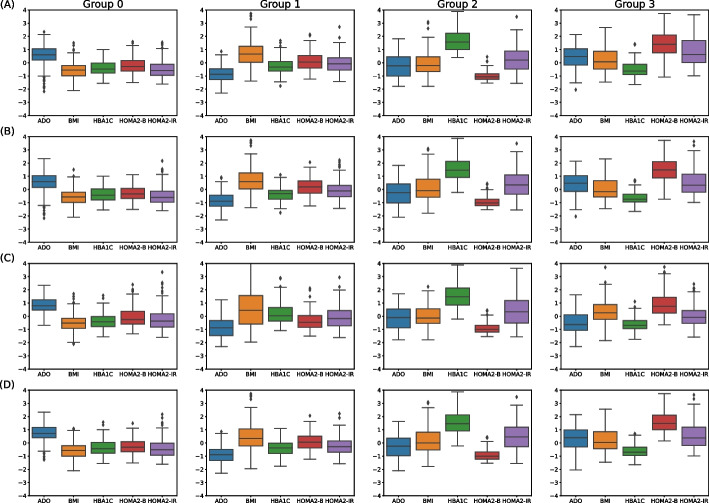
Box plots of the four implemented clustering strategies in first stage clustering. (**A**) to (**D**) correspond to strategies 1.1 to 1.4, in that order

The comparison among the first stage clustering strategies is provided in Table 3. It can be observed how the similarities among clusterings provide further means to evaluate them. The best validated clustering 1.4 attained good similarities with clustering strategies 1.1 and 1.2. On the contrary, strategy 1.3 yielded a rather dissimilar grouping with respect to its counterparts, even on this relatively small dataset. In addition to these results, Fig. 3 contains box plots showing the distribution of attributes per group, for each of the four implemented clustering strategies. Groups of the four strategies were identified and changed to match by observing the corresponding pattern in the plots. The order of attributes per group is the same: ADO, BMI, HbA$_\text {1C}$, HOMA2-B, and HOMA2-IR. As it is apparent from these plots, strategies 1.1, 1.2, and 1.4 also yielded similar clusters. It is also noticeable that the distribution of attributes of clustering 1.3 did not suit the rest of them, particularly in groups 1 and 3.

**Table 3:** Comparison metrics for first stage clustering. Dataset $D_2$ (*N* = 680). *ARI* adjusted rand index, *AMI* adjusted mutual index, *FM* Fowlkes-Mallows index. Best metric value achieved appears in bold

Strat. *i*	Strat. *j*	ARI	AMI	FM
1.1	1.2	**0.8139**	**0.7629**	**0.8659**
1.1	1.3	0.4302	0.4073	0.5885
1.1	1.4	0.7011	0.6658	0.7838
1.2	1.3	0.4440	0.4365	0.5965
1.2	1.4	0.7530	0.7363	0.8205
1.3	1.4	0.4866	0.4778	0.6261

Based on these first stage clustering results, we chose strategies 1.2 and 1.4 and computed centroids either for the whole clustering (strategies 2.1 and 2.3, respectively) and for clusterings separated by gender (strategies 2.2 and 2.4, respectively). Additionally, we performed repeated K-means procedures for CP-HOMA2 and IN-HOMA2 attributes, the latter using *Min-Max* normalization instead of *z-score* (strategies 2.5 and 2.6, respectively). Table 4 summarizes results from second stage clustering. Overall, group proportions were similar across all the strategies, being Group 0 the majority group with proportions ranging from 39.4 to 43.2%. Groups 1, 2, and 3 showed almost identical proportions in strategies 2.1 to 2.5. These percentages ranged from 18.9 to 21.1%, 18.5 to 19.9%, and 19.2 to 20.7%, respectively, for Groups 1, 2, and 3. On the other hand, clustering 2.6 generated slightly different populated clusters with proportions of 17.6, 16.3, and 22.8%, respectively, in Groups 1, 2, and 3. In terms of clustering validation indices, both strategies implemented with repeated K-means procedure outperformed those with centroid initialization. Moreover, clustering 2.6, achieved notably better metric scores than its nearest competitor strategy 2.5. Also, comparing strategies with initial centroids, it is observable that those without gender separation (2.1 and 2.3) obtained better scores than their gender-separated counterparts.

**Table 4:** Results for second stage clustering. Dataset $D_1$ (*N* = 2,768). *SIL* silhouette, *DB* Davies-Bouldin, *CH* Calinski-Harabasz. Best metric value achieved appears in bold

	Obs. per group				Intrinsic index		
Strat.	0	1	2	3	SIL	DB	CH
2.1	1160	523	511	574	0.1976	1.5518	629.61
2.2	1140	555	542	531	0.1883	1.6004	607.14
2.3	1138	546	511	573	0.1959	1.5530	631.39
2.4	1092	584	552	540	0.1798	1.6188	601.69
2.5	1155	530	549	534	0.2118	1.4701	671.79
2.6	1196	488	452	632	**0.2580**	**1.2120**	**934.23**

Table 5 shows comparison metrics obtained for all-pairs of the six clustering strategies implemented in the second stage clustering. Interestingly, the pair of strategies (2.1, 2.3) attained the highest similarity scores, despite that they originated from different first stage centroids. These scores were substantially higher, even compared with that of the pairs (2.1, 2.2) and (2.3, 2.4), that originated from the same first stage clusterings 1.2 and 1.4, respectively. Moreover, the second similar pair was (2.2, 2.4), which also come from different centroid initialization. Pairs of strategies that come from the same first stage clusterings (i.e. (2.1, 2.2) and (2.3, 2.4)) obtained the third and fourth places in terms of these clustering validity metrics. The remaining of clustering pairs that used *z-score* normalization and CP-HOMA2 values (2.1 to 2.5) reached scores ranging from: 0.6538 to 0.7512 (ARI), 0.6122 to 0.6853 (AMI), and 0.7517 to 0.8221 (FM). Finally, all comparison pairs involving the clustering 2.6 that used IN-HOMA2 values with *Min-Max* normalization, obtained lower score ranges: 0.3289-0.3784 (ARI), 0.3380-0.3828 (AMI), and 0.5250-0.5533 (FM).

**Table 5:** Comparison metrics for second stage clustering. Dataset $D_1$ (*N* = 2,768). *ARI* adjusted rand index, *AMI* adjusted mutual index, *FM* Fowlkes-Mallows index. Best metric value achieved appears in bold

Strat. *i*	Strat. j	ARI	AMI	FM
2.1	2.2	0.8065	0.7396	0.8619
2.1	2.3	**0.9379**	**0.9099**	**0.9557**
2.1	2.4	0.7269	0.6803	0.8042
2.1	2.5	0.7362	0.6563	0.8121
2.1	2.6	0.3289	0.3380	0.5250
2.2	2.3	0.7512	0.6853	0.8221
2.2	2.4	0.8449	0.7969	0.8886
2.2	2.5	0.7298	0.6577	0.8070
2.2	2.6	0.3481	0.3556	0.5375
2.3	2.4	0.7768	0.7288	0.8396
2.3	2.5	0.7023	0.6340	0.7874
2.3	2.6	0.3389	0.3415	0.5310
2.4	2.5	0.6538	0.6122	0.7517
2.4	2.6	0.3734	0.3696	0.5533
2.5	2.6	0.3435	0.3828	0.5350

**Fig. 4:**
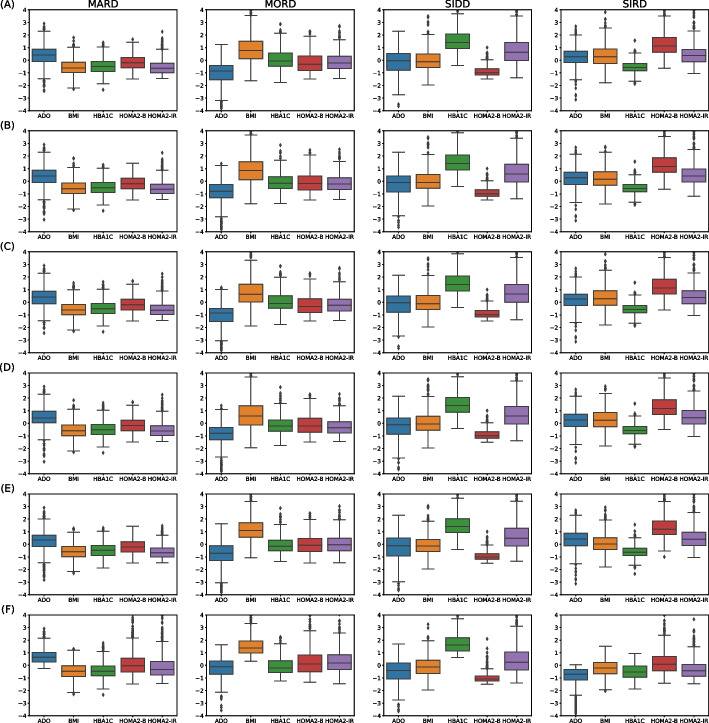
Box plots of the six implemented clustering strategies on dataset $D_1$ (*N* = 2,768). Panels (**A**) to (**F**) corresponds to strategies 2.1 to 2.6, in that order

Figure 4 shows the distribution patterns of involved attributes for the six clustering strategies applied on dataset $\text {D}_1$. The order of attributes per Group is the same: ADO, BMI, HBA1C, CP-HOMA2-%$\beta$, and CP-HOMA2-IR. Importantly, these distribution plots allowed us to assign T2DM subtype to each cluster, by means of visual inspection and direct comparison of the patterns against previous results in T2DM sub-classifications [8, 9, 30]. Indeed, the patterns of attributes obtained within the different clusters matched the distributions previously reported for MARD, MORD, SIDD, and SIRD. In general, patterns from all six clustering strategies were sufficiently matching to that of previously reported in the literature to distinguish and assign a T2DM subtype to each group. Nevertheless, as it is observable on the plots, there are some slight differences in ranges, interquartile ranges, and outliers comparing distribution of attributes in the T2DM subtypes. Among these minor discrepancies, the most appreciable were (see Fig. 4): both HOMA2 values in MARD (Panels A-E compared to F); BMI in MORD (Panels A-D compared to E and F); HBA1C in SIDD (Panels A-E compared to F); ADO and HBA1C in SIRD (Panels A-D compared to E and F).

From the second stage clustering on dataset $\text {D}_1$, and considering validation and comparison metrics, we selected the groups produced by two clustering strategies to constitute two labeled datasets: *Dset A* and *Dset B*, from strategies 2.5 and 2.6, respectively. On these datasets, T2DM subtype labels were assigned to patients by means of the matching of patterns in the identified groups by clusterings 2.5 and 2.6 (Panels (E) and (F) in Fig. 4). Both datasets were used in the next stage for developing classification models.

### Obtaining classification models

Based on the labeled datasets *Dset A* and *Dset B*, the four classification algorithms learned about these T2DM subtypes. Models trained with both datasets are presented in Table 6. For brevity, we will refer to them as models A and B. Each entry in Table 6 displays global results that include median accuracies (ACC) and median weighted-averaged F1-scores (F1) with respective 95% CI computed from bootstrap validation (1000 samples) for each of the seven data schemes and four algorithms implemented. In our discussion, we will use the term “best performing” algorithm/model referring to the one that achieved the highest median/mean metric value, regardless of having overlapping confidence intervals.

**Table 6:** Bootstrap validation results. Global classification metrics obtained for models A and B. Median accuracies (ACC) and F1-scores (F1) are presented with respective 95% CI. Best performing model on each scheme appears in bold

		Models A		Models B	
Scheme	Algorithm	ACC (95% CI)	F1 (95% CI)	ACC (95% CI)	F1 (95% CI)
S1	SVM	0.9862 (0.978–0.993)	0.9862 (0.978–0.973)	0.9794 (0.969-0.987)	0.9794 (0.969-0.987)
	KNN	0.9292 (0.912–0.947)	0.9284 (0.910–0.946)	0.9307 (0.910-0.947)	0.9298 (0.909-0.947)
	MLP	**0.9880 (0.981–0.994)**	**0.9880 (0.981–0.994)**	**0.9832 (0.972-0.991)**	**0.9832 (0.971-0.991)**
	SNNN	0.9462 (0.928–0.962)	0.9463 (0.928-0.962)	0.8177 (0.782-0.845)	0.8053 (0.759-0.836)
S2	SVM	**0.8271 (0.807–0.846)**	**0.8232 (0.802–0.843)**	0.9835 (0.974-0.990)	0.9835 (0.975-0.990)
	KNN	0.8074 (0.785–0.828)	0.8023 (0.778–0.824)	0.9542 (0.938-0.968)	0.9539 (0.937-0.968)
	MLP	0.8166 (0.795–0.836)	0.8154 (0.793–0.835)	**0.9891 (0.979-0.995)**	**0.9891 (0.979-0.995)**
	SNNN	0.8131 (0.788–0.837)	0.8106 (0.782–0.836)	0.8037 (0.766-0.830)	0.7871 (0.729-0.819)
S3	SVM	**0.7801 (0.759–0.803)**	**0.7762 (0.753–0.800)**	0.8927 (0.876-0.908)	0.8908 (0.874-0.907)
	KNN	0.7735 (0.752–0.797)	0.7687 (0.746–0.794)	0.8751 (0.856-0.893)	0.8735 (0.853-0.891)
	MLP	0.7643 (0.742–0.786)	0.7625 (0.740–0.784)	**0.8934 (0.876-0.909)**	**0.8917 (0.874-0.908)**
	SNNN	0.7777 (0.750–0.802)	0.7760 (0.744–0.801)	0.7446 (0.705-0.774)	0.7287 (0.659-0.763)
S4	SVM	0.9613 (0.949–0.972)	0.9611 (0.949–0.972)	0.9788 (0.969-0.987)	0.9788 (0.969-0.987)
	KNN	0.8921 (0.872–0.911)	0.8902 (0.870–0.910)	0.9037 (0.882-0.924)	0.9023 (0.881-0.924)
	MLP	**0.9781 (0.968–0.986)**	**0.9781 (0.968–0.986)**	**0.9833 (0.971-0.991)**	**0.9833 (0.971-0.991)**
	SNNN	0.9041 (0.882–0.922)	0.9044 (0.882–0.922)	0.7770 (0.736-0.808)	0.7563 (0.689-0.792)
S5	SVM	**0.8222 (0.803–0.842)**	**0.8177 (0.797–0.839)**	0.9772 (0.968-0.985)	0.9772 (0.968-0.985)
	KNN	0.7948 (0.773–0.818)	0.7863 (0.760–0.811)	0.8988 (0.877-0.919)	0.8974 (0.875-0.919)
	MLP	0.8149 (0.793–0.835)	0.8140 (0.792–0.834)	**0.9851 (0.974-0.992)**	**0.9851 (0.974-0.992)**
	SNNN	0.8049 (0.778–0.831)	0.8011 (0.769–0.828)	0.7740 (0.736-0.805)	0.7535 (0.684-0.791)
S6	SVM	0.7937 (0.763–0.819)	0.7808 (0.742–0.811)	0.9806 (0.971-0.988)	0.9806 (0.971-0.988)
	KNN	0.7553 (0.731–0.778)	0.7310 (0.698–0.759)	0.9504 (0.934-0.965)	0.9502 (0.933-0.964)
	MLP	**0.8190 (0.797–0.838)**	**0.8175 (0.796–0.837)**	**0.9810 (0.970-0.989)**	**0.9810 (0.970-0.989)**
	SNNN	0.7704 (0.742–0.795)	0.7608 (0.727–0.789)	0.9145 (0.848-0.956)	0.9127 (0.841-0.955)
S7	SVM	**0.7579 (0.737–0.781)**	**0.7379 (0.709–0.766)**	0.9771 (0.968-0.985)	0.9771 (0.968-0.985)
	KNN	0.7431 (0.720–0.766)	0.7216 (0.691–0.749)	0.9312 (0.914-0.948)	0.9306 (0.913-0.947)
	MLP	0.7398 (0.713–0.763)	0.7296 (0.705–0.754)	**0.9785 (0.966-0.987)**	**0.9785 (0.965-0.987)**
	SNNN	0.7455 (0.717–0.772)	0.7348 (0.693–0.763)	0.8062 (0.765-0.839)	0.7944 (0.727-0.831)

Naturally, as it consists of the same attributes on which *Dset A* was clustered, the best performance among models A was attained by scheme S1 with same ACC and F1 values of 98.8% (98.1–99.4% CI) . Nonetheless, the next best performing scheme (S4) was not far from these metrics reaching up to 97.8 (96.8–98.6% CI) ACC and F1. Remaining (best performing) models A produced ACCs ranging from 75.8 to 82.7% and F1s ranging from 73.9 to 82.3%. Algorithms that yielded the highest ACC were SVM (schemes S2, S3, S5, and S7) and MLP (schemes S1, S4, and S6). Moreover, these algorithms obtained the best and second-best performance in all schemes excepting S3 and S7, where KNN and SNNN attained the second-best performance, respectively. SVM kernels that performed best were *linear* (schemes S1, S3, S4, S6, and S7) and *rbf* (schemes S2 and S5). There were marginal differences among K values tested in KNN, with values K=54 and K=55 achieving the best in most of the schemes. Evaluating mean performance of best models across all seven schemes, mean ACC and F1 were 85.3% (± 9.2%) and 84.8% (± 9.7%), respectively.

Among models B, the best performing were also the ones from which the input dataset was labeled (in this case, scheme S2), with 98.9% (97.9–99.5% CI) of both best ACC and F1. However, in this case, the rest of models offered considerable closer performances with respect to S2, in all schemes excepting S3. Indeed, second to sixth performing models (schemes S5, S4, S1, S6, and S7) achieved ACCs and F1s ranging from 97.9 to 98.5% (i.e. only 1.0 to 0.4% lower than S2), while S3 attained lower ACC = 89.3% and F1 = 89.2%. In this case and within all schemes, MLP outperformed the rest of algorithms closely followed by SVM, particularly in schemes S6 and S7. Interestingly enough, SVM kernel that produced best results was *polynomial* within these models. Again, tested K values did not yield substantial difference in performance for models B. The mean performance of best models in all schemes is given by ACC and F1 values of 97.1% (± 3.4%) and 97.0% (± 3.5%), respectively.

Supplementary Tables S1 and S2 show corresponding per-class results of models A and B, respectively, in terms of F1-score, Sensitivity, and Specificity. In these tables, each entry displays the metrics for the best performing model (i.e. best ACC), out of the 1000 bootstrap samples. Corresponding confusion matrices from which these metrics were computed are also included in Supplementary Figs. S1 and S2. By observing Table S1 and corresponding Fig. S1, it can be noticed that the lower performance of models A within schemes S2, S3, S5, S6, and S7 is mainly due to a poor Sensitivity for Class 3 (SIRD). This metric was drastically low in schemes S6 and S7 where some algorithms reached values even lower than 40%. This effect is evidenced in the confusion matrices by observing that most errors come from Class 3 cases being misclassified as Class 0, and vice versa. Interestingly, that was not the case for models B (Table S2). In these models, the abnormal low sensitivities occurred only in Class 1 (MORD) and only for SNNN. This result is also explained by watching that many Class 1 records are misclassified as Class 0, 2, or 3 (Fig. S2) in most of schemes.

The amounts of records of each class left in the *out-of-bag* (validation) set are also shown in Tables S1 and S2. It can be observed that the proportion of validation records from the input dataset is $\sim$ 35–38% in these samples. This means that the models were trained using a proportion of $\sim$ 62–65% of different records from the input dataset. In other words, 35 to 38% of the training records are repeated in the bootstrap process.

**Fig. 5:**
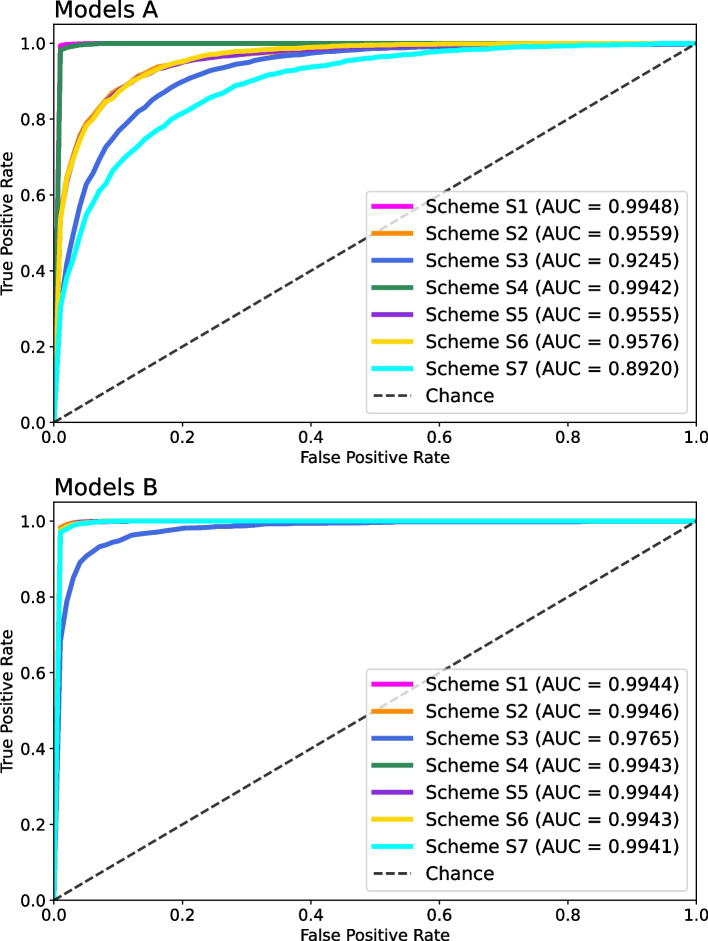
Macro-averaged Receiver Operating Characteristics curves for each scheme. (**A**) Models A. (**B**) Models B

For this reason and with the purpose of contrasting results with those reported by [9], we also aimed at assessing the performance of classification models A and B using a stratified 10-fold cross validation. We selected the best performing algorithm in each scheme from the bootstrap validation stage; as reviewed above (i.e. those appearing bolded in Table 6). Table 7 shows these classification results computed as the mean values across the 10 folds for global Accuracy and per-class Precision, Sensitivity, Specificity, and Area Under the Curve (AUC). The overall performance of all models was consistent compared with bootstrap results, with minor increases and decreases in ACC. For models A, it is noticeable the same behavior observed in bootstrap regarding the low sensitivity in Class 3 for schemes S2, S3, S5, S6, and S7. With respect to implemented schemes S1, S2, S3, and S7 in [9], our models A achieved comparable performance in S1, but yielding lower metric values in the rest of them. Conversely, models B produced remarkable competitive performances in all compared schemes. Lastly, Fig. 5 compares macro-averaged Receiver Operating Characteristics (ROC) curves and displays corresponding AUCs for both models A and B, and for each of the seven implemented schemes. In the case of models A (upper panel), these plots show how schemes S1 and S4 attained the best performance, with considerable higher AUC than the rest of schemes. For models B (lower panel), it can be observed that excepting for S3, all schemes obtained closely similar curves and AUC values.

**Table 7:** Stratified 10-fold cross-validation results. Global accuracy (ACC) with per-class precision (PRE), sensitivity (SEN), specificity (SPE), and area under the curve (AUC) are shown for models A and B; and contrasted with those reported by [9]. Each entry of our results corresponds to the mean value obtained across the 10 folds. Only best performing algorithms from bootstrap validation were included (i.e. those appearing in bold from Table 6)

		Models A					Models B					[9]				
Scheme	Class	ACC	PRE	SEN	SPE	AUC	ACC	PRE	SEN	SPE	AUC	ACC	PRE	SEN	SPE	AUC
S1	MARD	0.990	0.991	0.993	0.994	1.000	0.982	0.989	0.989	0.992	0.999	0.981	1.000	0.987	1.000	1.000
	MORD		0.983	0.985	0.996	1.000		0.969	0.986	0.991	0.998		0.992	1.000	0.973	1.000
	SIDD		0.991	0.987	0.998	1.000		0.982	0.969	0.996	0.999		0.998	0.994	0.991	0.990
	SIRD		0.994	0.992	0.999	1.000		0.985	0.975	0.997	1.000		0.998	1.000	0.991	1.000
S2	MARD	0.832	0.824	0.894	0.863	0.945	0.988	0.990	0.996	0.992	1.000	0.903	0.945	0.876	0.914	0.880
	MORD		0.863	0.875	0.967	0.985		0.987	0.978	0.996	0.999		0.988	0.966	0.954	0.930
	SIDD		0.901	0.904	0.975	0.994		0.986	0.990	0.997	0.999		0.984	0.988	0.931	0.970
	SIRD		0.733	0.582	0.949	0.907		0.989	0.982	0.998	1.000		0.901	0.959	0.656	0.860
S3	MARD	0.760	0.734	0.902	0.764	0.898	0.899	0.937	0.964	0.951	0.990	0.859	0.912	0.856	0.891	0.840
	MORD		0.804	0.809	0.953	0.954		0.898	0.903	0.970	0.989		0.979	0.950	0.921	0.920
	SIDD		0.814	0.792	0.955	0.958		0.891	0.940	0.975	0.994		0.935	0.954	0.774	0.850
	SIRD		0.716	0.372	0.965	0.891		0.789	0.677	0.965	0.942		0.903	0.949	0.627	0.840
S4	MARD	0.979	0.985	0.991	0.989	0.999	0.985	0.990	0.993	0.992	0.999	–	–	–	–	–
	MORD		0.970	0.964	0.993	0.999		0.971	0.982	0.992	0.998		–	–	–	–
	SIDD		0.981	0.978	0.995	0.999		0.994	0.974	0.999	0.999		–	–	–	–
	SIRD		0.974	0.968	0.994	0.998		0.982	0.982	0.997	1.000		–	–	–	–
S5	MARD	0.833	0.824	0.877	0.865	0.943	0.987	0.992	0.994	0.994	0.999	–	–	–	–	–
	MORD		0.874	0.904	0.969	0.985		0.973	0.991	0.992	0.999		–	–	–	–
	SIDD		0.910	0.922	0.977	0.994		0.994	0.978	0.999	0.999		–	–	–	–
	SIRD		0.714	0.578	0.945	0.906		0.991	0.976	0.998	1.000		–	–	–	–
S6	MARD	0.825	0.829	0.861	0.873	0.948	0.983	0.987	0.992	0.990	0.999	–	–	–	–	–
	MORD		0.857	0.878	0.965	0.984		0.977	0.974	0.993	0.998		–	–	–	–
	SIDD		0.914	0.912	0.979	0.994		0.984	0.981	0.996	0.999		–	–	–	–
	SIRD		0.678	0.605	0.930	0.911		0.979	0.972	0.996	1.000		–	–	–	–
S7	MARD	0.738	0.715	0.904	0.738	0.881	0.982	0.991	0.990	0.993	0.999	0.820	0.923	0.878	0.884	0.880
	MORD		0.797	0.819	0.949	0.957		0.975	0.970	0.992	0.998		0.981	0.971	0.934	0.940
	SIDD		0.880	0.881	0.971	0.980		0.971	0.976	0.994	0.999		0.984	0.979	0.928	0.950
	SIRD		0.404	0.153	0.943	0.753		0.980	0.981	0.996	0.999		0.897	0.941	0.601	0.820

As a final step in the classification stage of our data analysis flow, we tested our trained models on unseen data. The hold-out dataset comprised *N* = 7,309 patient records that did not include C-peptide values and thus, was a disjoint set with respect to the training/validation dataset. As previously explained, we applied a majority vote approach using the best performing models A, considering the five schemes which did not make use of C-peptide parameter (i.e. S2, S3, S5, S6, and S7). Table 8 shows the number of records that were classified in each class by the five predictors. Despite of the fact that there were disparities in these amounts (i.e. predictor S5), in general, there was consensus among the five predictors. On 77.3% of the observations, all five or four of the predictors agreed on the resulting class. Moreover, the cases when three or more predictors agreed amounted to 97% of observations. The total of ties (cases where two pairs of predictors voted for two different classes) were 175 (2.4%) and were solved by simply assigning the class predicted by the predictor that achieved the best performance during the bootstrap validation stage.

**Table 8:** Number of records classified per class in the hold-out dataset for each of the five predictors considered

	Schemes				
Class	S2	S3	S5	S6	S7
MARD	3048	3058	4260	3317	2832
MORD	1225	1273	1982	1402	1314
SIDD	1593	1561	592	1555	1193
SIRD	1443	1417	475	1035	1812

**Fig. 6:**
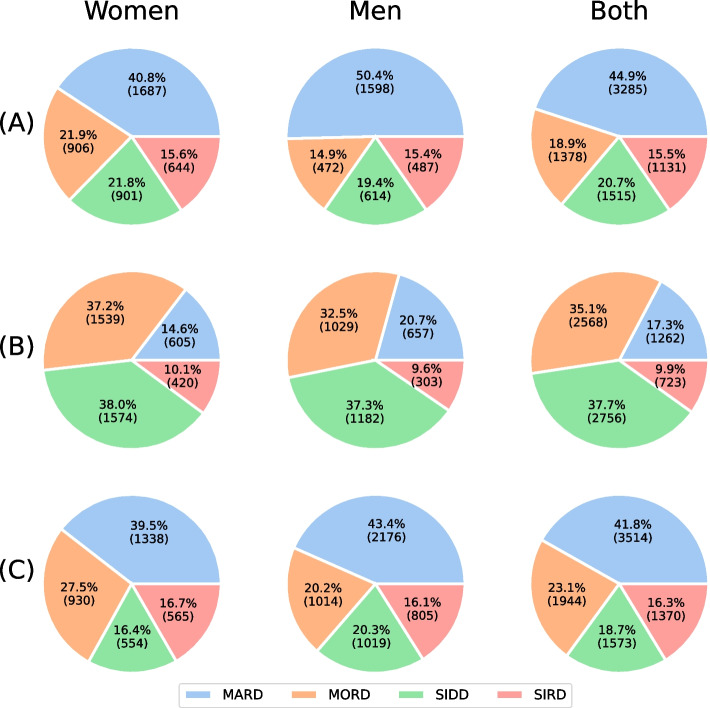
Proportion of observations of T2DM classes. (**A**) Our mayority vote scheme with models trained with $LD_1$ dataset. (**B**) Classification of test dataset using the insulin-based HOMA2 model developed by Bello-Chavolla et al. [9]. (**C**) Clustering results reported by Ahlqvist et al. [8] with their dataset ANDIS

Figure 6 depicts our final classification results (Panel A) on the test set in terms of the proportions of each class separated by gender or including both. For comparison purposes, we also include proportions obtained by landmark studies [8, 9]. The former (Panel B) were acquired by classifying our test set using the authors’ web tool with attributes corresponding to our scheme S2. The latter (Panel C) consist of the authors’ reported results obtained with a dataset of their own (ANDIS, Swedish population, *N* = 8,980). On the latter results, we recalculated the number of observations accordingly, after eliminating those belonging to the SAID class, which we did not consider. Proportions of classes from our majority vote approach were similar to that of [8], in spite of the fact that both were obtained from different populations. On the other hand, although the charts in Fig. 6 display different proportions with respect to [9], there was still an overall matching of 57.2% with 1152, 938, 1510, and 578 equally classified observations for MARD, MORD, SIDD, and SIRD, respectively. 90.3% of discrepancies came from observations that were respectively classified in our method/web tool as: MARD/MORD (1228), MARD/SIDD (790), MORD/SIDD (411), and SIRD/MORD (398).

Lastly, Fig. S3 shows a comparison of per-class distribution patterns for ADO, BMI, HBA1C, IN-HOMA2-%$\beta$, and IN-HOMA2-IR; for results obtained in the test set from our study (Panel A) and the aforementioned web classifier (Panel B). Overall, resemblance of patterns is appreciable for all variables, although, there was some variation derived from the disparities in amounts of observations per class. Due to the MARD/MORD and MARD/SIDD mismatching classifications, it is observable that the web classifier yielded a narrower distribution and higher median for ADO in MARD class; as this class has fewer instances. However, as a consequence of having more instances classified within, classes MORD and SIDD present less defined distributions of BMI and HBA1C, respectively.

## Discussion

In the present study, we have focused on developing and testing classification models for T2DM subtypes. Our methodology consisted in three main stages: *dataset construction*, *data characterization*, and *classification model development*. In view of our results, we consider the following as our findings.

First, producing an enriched large dataset by fusing information from two representative health databases, NHANES and ENSANUT. Although NHANES includes multi-ethnic information, our dataset predominantly comprised mexican-american, other hispanic, and mexican patients with approximately 60% of the total records. Thus, we consider that this dataset comprises a fairly representative sample of this population. Our dataset was amongst the largest within those related to application of unsupervised learning for diabetes [31].

Second, experimenting with more clustering algorithms such as density-based and hierarchical methods; and evaluating cluster qualities in terms of clustering validation indices. We verified that tested DBSCAN and Agglomerative algorithms did not yield good clusterings contrasted to K-means, according to intrinsic metrics; which, according to our knowledge, has not been reported by previous works. Also, amounts of observations within groups importantly differed from those obtained with K-means, as was corroborated by extrinsic metrics. Thus, on reported experiments we attached to previous proved methodologies that were based on K-means to characterize T2DM groups; on the basis that this unsupervised method provides the best means to find better defined and distinctive class boundaries. Additionally, we tested different clustering strategies contrasting centroid initialization, clustering by gender, and using a repeated K-means procedure. The latter simple procedure allowed us to deal with cluster variance within executions, occurring in some observations lying on an inter-cluster boundary. Results obtained in this stage suggest that better defined clusters are obtained by executing de novo K-means clustering and without gender separation.

And third, providing further insights of model performances in the classification of T2DM subtypes. In this regard, we carried out an exhaustive evaluation of four machine learning algorithms using two validation settings. Bootstrap is considered a more statistically robust way of assessing performance of machine learning models [52]. Nevertheless, both validation modes yielded similar results in terms of classification metrics applied. Interestingly, models fitted remarkably better to data that was clustered using *Min-Max* normalization and IN-HOMA2 measures, obtaining accuracies of 97.1 ± 3.4% (bootstrap) and 97.2 ± 3.2% (cross-validation), averaged from the seven implemented data schemes. These averaged accuracies were 85.3 ± 9.2% (bootstrap) and 85.1 ± 9.8% (cross-validation) in the case of models trained with *z-score* standardized data with CP-HOMA2. SVM and MLP machine learning techniques attained best performances. Above all, from the seven data schemes we assayed, we found that HOMA2 constituent variables (used in schemes S4 and S5) provided great performances. From our point of view this result was interesting, as it points that HOMA2 variables used for clustering can be replaced with surrogates to train classification models. Indeed, the importance of this finding lies on the fact that parameters such as fasting glucose and C-peptide/insulin are readily available from public databases or health records, while HOMA2 measures require licensed software when deploying tools in online production environments (although they provide offline converters free of access). To the best of our knowledge, with the exception of SNNN models [9, 39], development and testing of classification models for T2DM subtypes has not been previously reported in the literature.

Finally, our majority vote approach demonstrated a great deal of consensus amongst used classifiers, in the hold-out dataset. Class proportions were similar to those found in the pioneer study of Ahlqvist et al. [8]. On the other hand, we believe that the disparity in our results compared with those of the web classifier of Bello-Chavolla et al. [9] are mainly attributable to the standardization step. Indeed, during experimentation we encountered that this step, which depends on the distribution of variables in the dataset, greatly impacts classification results.

## Conclusion

We have introduced a new pipeline for analysis of datasets with the goal of obtaining classifiers for T2DM subtypes. With this purpose, we described a detailed data curation and characterization processes to obtained labeled datasets. Unlike previous work, our analysis included a clustering validation step through well-known indices, that allowed us to evaluate quality of clusters. We have obtained results consistent to most of previous work in terms of subgroup proportions (see Table 1). From the classifiers we have trained, it is remarkable the fact that simpler and faster algorithms such as SVM and MLP fitted better to the clustered data than the more involved convolutional architectures. Also, the results showed that classifiers learned better from normalized (*Min-Max*) compared to that of standardized (*z-score*) data. The obtained performances using this scaling approach were consistent across the seven data schemes, since normalized data produced better defined clusters according to validation indices.

The present work was based on cross-sectional data and thus, we have limited the scope of our analysis to the development of classification tools for T2DM subtypes, without further association with risks of complications, incidence, prevalence, and treatment response. We left such analyses as future work, with the hope of establishing data sharing collaborations. However, we believe that the study offers valuable insights on the process of developing classification models for T2DM subtypes. Further limitations of the present study are those inherent to the population (i.e. dataset) used for the analysis, preprocessing steps applied, and that we have considered all the patients within the dataset as GADA negative (i.e. not considering SAID class), since this variable was not available in most of NHANES and ENSANUT records.

### Supplementary Information

**Additional file 1.**

## Data Availability

The databases used for constructing datasets in this study are public and available to download from their sites [42, 43].

## Appendix A: Background definitions

This section will provide background concepts and definitions pertinent to the methods applied. In particular, we will describe clustering techniques, clustering validation indices, classification algorithms, and evaluation metrics.

### Clustering techniques

Clustering techniques differ on the way how clusters are identified. Basically, this depends on how the user desires the grouping approach and the distribution of data. Three different clustering approaches were explored:

- Hierarchical (*agglomerative clustering*) [45] is a bottom-up approach that begins grouping closer observations forming a tree (i.e. a hierarchy) termed dendrogram, where upper levels represents meta-clusters (cluster of clusters).
- Density (*DBSCAN* [46]) works on the density of instances into the space of instances, grouping those which are in dense regions to form clusters.
- Partitional (*K-means*) is directed by the number of desired clusters. Instances are grouped according to its similarity to the centroids of the clusters. K-means is one of the most used due to its simplicity and scalability. Given *k*, i.e. the desired number of clusters, k-means proceeds to coalesce instances by assigning them the closest centroid, based on some distance measure. Centroids are representative instances of clusters, initially given or pre-computed, that represent the center of each cluster located at the mean of each variable. Once every instance is assigned to its corresponding centroid, the next step consists on recomputing the *k* centroids. This process is repeated until the centroids do not change significantly according to a predefined tolerance value, or a maximum number of iterations is reached, or another stopping criteria.

### Clustering validation indices

Validation indices are mathematical formulations that provide quantitative measures for evaluating the quality of the clustering procedures. There are intrinsic and extrinsic methods. These are briefly described in the following.

#### Intrinsic methods

When the ground truth labels of instances are not available, intrinsic methods allow to quantify the quality of clusterings. The general idea consists in minimizing distances of instances within the same partition (i.e. obtain more compact partitions), and maximizing distances of observations belonging to different partitions (i.e. obtain more separation among partitions). The methods we applied were:

- **Silhouette (SIL)** [53]. The Silhouette index computes for each instance $p_i$ a score $\text {SIL}_i$, given by $\text {SIL}_i=(b_i-a_i)/\text {max}(b_i, a_i)$, where $a_i$ is the average distance of $p_i$ to every instance within its cluster and $b_i$ is the average distance of $p_i$ to all instances of the nearest cluster. The overall index for a clustering *C*, $\text {SIL}_C$ is obtained by averaging the index of all instances.
- **Davies-Bouldin (DB)** [54]. This index is defined as the average similarity between each cluster $C_i$ ($1\le i\le k$) and its most similar $C_j$. For each cluster $C_i$ let $R_{ij}=((s_i+s_j)/d_{ij})$ be this measure of similarity, where $s_i$, $s_j$ are respectively the average distance of each instance in $C_i$ to its centroid, and $d_{ij}$ the distance between centroids *i* and *j*. The Davies-Bouldin index is defined as the average of the similarity between clusters $C_i$ and $C_j$:
  $$\begin{aligned} DB=\frac{1}{k}\sum _{i=1}^{k}\max _{i\ne j}(R_{ij}) \end{aligned}$$
- **Calinski-Harabasz (CH)** [55]. This index is also known as the *Variance Ratio Criterion*. For a k-clustering of a dataset with *N* instances, the between- and within-cluster dispersion matrices are respectively defined as:
  $$\begin{aligned} B_K=\sum _{k=1}^{K}n_k(c_k-c_N)(c_k-c_N)^{T} \end{aligned}$$
  $$\begin{aligned} W_K=\sum _{k=1}^{K}\sum _{p\in C_k}(p-c_k)(p-c_k)^{T} \end{aligned}$$
  where $n_k$ and $c_k$ are the number of instances and centroid of the *k*-th cluster $C_k$ and $c_N$ is the global centroid of the dataset. The Calinski-Harabasz index is defined as the ratio
  $$\begin{aligned} CH=\frac{trace(B_k)}{trace(W_k)}\times \frac{N-K}{K-1} \end{aligned}$$

#### Extrinsic methods

On the other hand, the extrinsic methods assist in evaluation of clustering quality only respective to a ground truth label assignment, and without considering any other information of distance among the data points. We used the following indices with the purpose of comparing similarity among our different clustering strategies:

- **Adjusted Rand Index (ARI)** [56]. For a ground truth label assignment *C* and a clustering *K*, let us define *a*, *b*, *c*,  and *d*, respectively as the number of pairs of instances that:
  - are in the same set in *C* and in the same set in *K*,
  - are in the same set in *C* and in different sets in *K*,
  - are in different sets in *C* and in the same set in *K*,
  - are in different sets in *C* and in different sets in *K*.
  Terms *a*, *b*, *c*,  and *d* can be calculated from the *contingency matrix* [57]. The unadjusted *Rand Index* is defined by:
  $$\begin{aligned} RI=\frac{a+b}{a+b+c+d} \end{aligned}$$
  To guarantee that random label assignments will get a value closer to zero, the *Adjusted Rand Index* is defined as:
  $$\begin{aligned} ARI=\frac{\left( {\begin{array}{c}N\\ 2\end{array}}\right) (a+d)-[(a+b)(a+c)+(c+d)(b+d)]}{\left( {\begin{array}{c}N\\ 2\end{array}}\right) ^{2}-[(a+b)(a+c)+(c+d)(b+d)]} \end{aligned}$$
- **Adjusted Mutual Information (AMI)** [58]. Let *U* and *V* be two label assignments for *N* instances in a clustering. Let us define the probability that a randomly picked instance falls into:
  - class $$U_i$$ as $$P(i)=\varvec{|} U_i \varvec{|}/N$$,
  - class $V_j$ as $P'(j)=\varvec{|} V_j \varvec{|}/N$, and
  - both classes $U_i$ and $V_j$ as $P(i,j)=\varvec{|} U_i\cap V_j \varvec{|}/N$.
  The entropy (amount of uncertainty) of each assignment are defined as:
  $$\begin{aligned} H(U)= -\sum _{i=1}^{|U|}P(i)\text {log}(P(i)) \end{aligned}$$
  $$\begin{aligned} H(V)= -\sum _{j=1}^{|V|}P'(j)\text {log}(P'(j)) \end{aligned}$$
  The unadjusted *Mutual Information* score for *U* and *V* is:
  $$\begin{aligned} MI(U,V)=\sum _{i=1}^{|U|}\sum _{j=1}^{|V|}P(i,j)\text {log}(\frac{P(i,j)}{P(i)P'(j)}), \end{aligned}$$
  and with the expected value for MI *E*(*MI*) the *Adjusted Mutual Information* score is defined as:
  $$\begin{aligned} AMI=\frac{MI(U,V)-E[MI]}{\text {mean}(H(U),H(V))-E[MI]}. \end{aligned}$$
- **Fowlkes-Mallows (FM)** [59]. This index is defined as the geometric mean of the pairwise *precision* and *recall* metrics. In notation of terms *a*, *b*, *c*,  and *d*, defined previously for the *ARI* index the *FM* score is defined as:
  $$\begin{aligned} FM=\frac{a}{\sqrt{(a+b)(a+c)}} \end{aligned}$$

### Classification algorithms

Algorithms used for developing classification models were *K-Nearest Neighbors* (K-NN), *Support Vector Machine* (SVM), *MultiLayer Perceptron* (MLP), and *Self-Normalized Neural Networks* (SNNN). These are briefly described in the following.

- **K-NN.** It is one of the simplest algorithm for classification. It is based on two simple notions, a measure of distance and the premise that closeness among patients is helpful to infer its class (T2DM subtype) membership [60]. Classification is made in two basic steps, first find the *K* nearest neighbors of an input patient and then classify the patient on a majority vote basis. It is called a lazy learning method in the sense that does not perform a training like other methods but rather classifies new patients using the data itself.
- **SVM.** This algorithm finds the best hyperplane (*support vector*) that best separates each class in the input data. In the binary case, the objective is to find a hyperplane that has the maximum distance between instances of a pair of classes. This maxim margin distance is useful so that a future instance can be classified. In case that the distribution of the classes in the training data is not lineally separable, it is necessary to modify the dimensionality of the data via a *kernel* function. In some cases, it is necessary to try different kernel functions to find the most suitable one.
- **MLP.** This is one of the simplest neural networks, but powerful for classification because it can learn linear and non-linear relations in data. The input data helps is combined to adjust a set of initial weights and bias arranged into layers, each linear combination in a layer is propagated to the next layer. By this, the model learns a set of patterns that describe the input data of each class. It can use any arbitrary activation function at the output. By several iterations on the input data, the algorithm readjusts weights and learning rate until no improvement is noticed in the classification. The resulting model classifies unseen patients.
- **SNNN.** This is a kind of deep learning technique; at first, this was defined as a new architecture of neural networks, but later it was defined as a variant of MLP. Its main feature is an implementation of the *SELU* (*Scaled Exponential Linear Units*) activation function. Neuron activations converge towards zero mean and unit variance even under the presence of perturbations in data. In this way, the data is self-normalized as it passes by each layer of the network making learning highly robust.

Values for *K* in K-NN were selected in the neighborhood of $\lfloor \sqrt{N} \rfloor$ (i.e. the interval $[\lfloor \sqrt{N} \rfloor -3, \lfloor \sqrt{N} \rfloor +3]$, where *N* is the number of patients. The hyperparameters for SVM, MLP, and SNNN were selected on preliminary execution of algorithms using grid search.

### Classification metrics

Each of the obtained data models was evaluated by means of the following multi-class metrics. For an *M*-class classification problem with *N* instances, let us consider the $M\times M$ confusion matrix CONF = $c_{ij}$, where by convention, we put the actual labels (*ground truth*) in columns and predicted labels in rows. For the *k*-th class $1\le k\le M$:

- True Positives ($\text {TP}_k$) are in position $c_{ij}$ ($i=k, j=k$)
- True Negatives ($\text {TN}_k$) are given by $\sum c_{ij}$ ($i\ne k, j\ne k$)
- False Positives ($\text {FP}_k$) are given by $\sum c_{ij}$ ($i=k, j\ne k$)
- False Negatives ($\text {FN}_k$) are given by $\sum c_{ij}$ ($i\ne k, j=k$)

With these values the per-class metrics Precision (PRE), Sensitivity or Recall (REC), their harmonic mean termed F1-score (F1), and Specificity (SPE) are respectively defined by:

$$\begin{aligned} \text {PRE}_{k}=\frac{\text {TP}_{k}}{\text {TP}_{k}+\text {FP}_{k}}, \end{aligned}$$

$$\begin{aligned} \text {REC}_{k}=\frac{\text {TP}_{k}}{\text {TP}_{k}+\text {FN}_{k}}, \end{aligned}$$

$$\begin{aligned} \text {F1}_{k} = 2\times \frac{\text {PRE}_{k}\times \text {REC}_{k}}{\text {PRE}_{k}+\text {REC}_{k}} = \frac{2\text {TP}_{k}}{2\text {TP}_{k}+\text {FP}_{k}+\text {FN}_{k}}, \end{aligned}$$

$$\begin{aligned} \text {SPE}_{k}=\frac{\text {TN}_{k}}{\text {TN}_{k}+\text {FP}_{k}}. \end{aligned}$$

Global PRE, REC, and F1 are computed by averaging or adding up per-class values. These forms of averaging are:

1. *macro-averaged*, which is the direct average of all classes (e.g. $\text {PRE}_{\textit{macro}} = (\text {PRE}_1+\text {PRE}_2+\dots +\text {PRE}_M)/M$);
2. *weighted-averaged*, which takes the proportion of instances on each class as weights (e.g. $\text {PRE}_{\textit{weighted}} = w_1\text {PRE}_1+w_2\text {PRE}_2+\dots +w_M\text {PRE}_M$, with $w_k=N_k/N$ for $1\le k\le M$); and
3. *micro-averaged*, which follows the one-vs-rest approach pooling the results of the $M\times M$ confusion matrix into a binary $2\times 2$ matrix, where TP, TN, FP, and FN values are the sum of its corresponding per-class values (e.g. TP = $\text {TP}_1+\text {TP}_2+\dots +\text {TP}_M$). Micro-averaged PRE, REC, and F1 turn out to be the same that the overall Accuracy (ACC) value, which in the multi-class setting is given by:
  $$\begin{aligned} \text {ACC}=\frac{\sum _{k=1}^{M}\text {TP}_{k}}{N}. \end{aligned}$$

## Appendix B: Data preparation

Data preparation/preprocessing is a crucial stage in a machine learning pipeline. Here, we will describe in detail the data transformation steps we carried out to construct our dataset. For the implementation of these steps we used *Pandas* and *Sklearn* libraries, versions 1.4.2 and 1.0.2, respectively.

### Data merging

After downloading the appropriate files/tables containing the variables needed for our study, we converted the format of files to *.CSV* when it was necessary. Particularly, we used the Python *XPORT* library to convert NHANES SAS *.XPT* files. Afterwards, we first merged tables within each cycle separately, and then concatenated the partial dataset obtained from each cycle. The set of attributes we initially selected from databases are shown in Table 9. To deal with variable naming inconsistencies across different cycles, we defined an ordering and naming. For some categorical variables (e.g. ETHNICITY or DIABETES) we also defined a consistent scheme of values and changed them accordingly. All values that were identified in the database documentation as codes for missing or irrelevant data were set to zero. Each observation was identified with a new sequential variable “NSEQN” and a cycle identifier “CYCLE” was also added. Table 10 resumes this information about each cycle.

**Table 9:** Subset of selected attributes

Attribute	Description
Age	Patient age in years
Gender	Patient gender
Ethnicity	Patient ethicity (Mex-Amer, hispanic, white, black, other)
Diabetes	Diabetes status (NA, diabetic, non-diabetic, borderline, gestational, etc.)
ADO	Age at Diabetes Onset in years
BMI	Body Mass Index (kg/m$^2$)
Height	Height in cm
Weight	Weight in kg
Waist	Waist circumference in cm
HbA1C	Glycated Haemoglobin as percentage
Glucose1 (FPG)	Fasting plasma glucose in mmol/L
Insulin	Fasting plasma insulin in pmol/L
C-peptide	Fasting plasma C-peptide in nmol/L
Glucose2 (OGTT-PG)	Plasma glucose after Oral Glucose Tolerance Test (OGTT) in mmol/L
HDLC	High-Density Lipoprotein Cholesterol in mmol/L
Triglyceride	Triglyceride in mmol/L

**Table 10:** Datasets generated per cycle. Cycles 1-11 are from NHANES and cycles 21-23 are from ENSANUT

CYCLE	Years	NSEQN	Size
1	1988-1998	0 - 20049	20050
2	1999-2000	20050 - 30014	9965
3	2001-2002	30015 - 41053	11039
4	2003-2004	41054 - 51175	10122
5	2005-2006	51176 - 61523	10348
6	2007-2008	61524 - 71672	10149
7	2009-2010	71673 - 82209	10537
8	2011-2012	82210 - 91965	9756
9	2013-2014	91966 - 102140	10175
10	2015-2016	102141 - 112111	9971
11	2017-2020	112112 - 127671	15560
21	2006	300000 - 345240	45241
22	2016	345241 - 354064	8824
23	2018	345065 - 397134	43070
		TOTAL	224807

### Data cleansing

Data cleansing steps consisted in a double-checking variable-per-variable for null/blank or coded values that were set to zero. In this step we also corrected data inconsistencies (e.g. some observations with AGE < ADO). Values that were declared in the database documentation as “below the limit of detection” (C-PEPTIDE and INSULIN in cycles 1 and 4) were also set to zero.

### Imputation

Prior to imputation, we maintained only adult patients (AGE $\ge$ 20, $N=172,909$) to avoid including young diabetic patients that are often of type I. Imputation was implemented in six incremental stages. For each imputed variable, we used four *Sklearn* estimator methods:

1. *Bayesian Regression*. It uses the available data to train a Bayesian ridge regression model to infer the missing data. By using a ridge approach, the resulting regression is intentionally offset from the original data to avoid overfitting the model.
2. *Decision Tree Regression*. It splits the existing data in several ranges per each row, depending on the ranges the predictor variables are, the outcome variable will be the mean of the rest of the data in the same range.
3. *Extra Trees Regression*. It works in a similar way to Decision Tree Regression, but instead of making rigorous calculus to find the optimal group splitting, a random splitting is performed.
4. *KNN Regression*. It uses the K-NN approach where the weighted mean of the k-nearest neighbours to the existing values in the row to impute are calculated to fill in the blanks.

We used the median of the four estimated values for each imputed variable. Estimators require to provide the dataset to impute and the dataset to fit, both with the same variables. *Pandas* functionality allow logical formulae with operator symbols “$\sim$, $\varvec{|}$, &” (NOT, OR, and AND; respectively) to be provided as queries to retrieve subsets of observations in a dataset. For instance, if define $v_1, v_2, v_3,$ and $v_4$ as the result of queries where variables satisfy some conditions (in our case, check if $v_1, v_2, v_3,$ and $v_4$ are present), then the query $v_1\ \varvec{|}\ (v_2$ & $\sim v_3)$ retrieves the dataset where either $v_1$ is present or $v_2$ is present and $v_3$ is absent. Using this notation, in the following we describe the six stages of our imputation scheme by providing the dataset to impute. We also provide the amount of observations in each dataset. In stages 1-4 we imputed observations separated by gender, and thus, the amounts are depicted as $N_m$ (men) and $N_w$ (women). For notation brevity, we will use the first two letters of each of the involved variables WEIGHT, HEIGHT, WAIST, HBA1C, GLUCOSE1, INSULIN, ADO, AGE, and BMI.

- Stage 1. Dataset to impute ($N_m=2380, N_w=3887$): AG & [($\lnot$HE & WE & WA) $\varvec{|}$ (HE & $\lnot$WE & WA) $\varvec{|}$ (HE & WE & $\lnot$WA)]. Dataset to fit ($N_m=55319, N_w=69051$): AG & HE & WE & WA.
- Stage 2. Dataset to impute ($N_m=130, N_w=160$): AG & [($\lnot$HE & WE) $\varvec{|}$ (HE & $\lnot$WE)]. Dataset to fit ($N_m=57699, N_w=72938$): AG & HE & WE.
- Stage 3. Dataset to impute ($N_m=130, N_w=160$): AG & HE & WE & $\sim$WA. Dataset to fit ($N_m=57699, N_w=72938$): AG & HE & WE & WA.
- Stage 4. Dataset to impute ($N_m=3974, N_w=6338$): AG & BM & [($\sim$HB & GL & IN) $\varvec{|}$ (HB & $\sim$GL & IN) $\varvec{|}$ (HB & GL & $\sim$IN)]. Dataset to fit ($N_m=26191, N_w=31288$): AG & BM & HB & GL & IN.
- Stage 5. Dataset to impute ($N=61037$): AG & HB & GL & IN & $\sim$AD. Dataset to fit ($N=7406$): AG & HB & GL & IN & AD.
- Stage 6. Dataset to impute ($N=24398$): AG & HB & $\sim$AD. Dataset to fit ($N=72216$): AG & HB & AD.

### Selection and extreme value removal

In this stage we selected patients that met the eligibility criteria, that is, patients with variables DIAGNOSED = 1 (diabetic) or with HBA1C $\texttt {>}$ 6.5% or GLUCOSE2 $\ge$ 200 mg/dl (glucose after oral glucose tolerance test, where this data was available). After selection, the size of the dataset was $N=21200$. We further selected observations that included values for C-PEPTIDE ($N=2889$) and carried out an extreme value removal procedure: observations with values that were separated from their mean for more than five standard deviations. We considered the variables ADO, BMI, GLUCOSE1, C-PEPTIDE, and INSULIN, and repeated the procedure until there were no more extreme values in neither of the variables ($N=2816$).

### HOMA2 computation

HOMA2 values which are derived from GLUCOSE1 and either C-PEPTIDE or INSULIN were computed using the excel version of the HOMA2 calculator downloaded from the authors’ webpage [48]. This calculator has some limit restrictions on the values accepted. We dealt with these restrictions by assigning the limit values when necessary. After the HOMA2 computation, we performed a second extreme value removal procedure based only on the HOMA2 (from C-peptide) variables, from which we obtained a final dataset with $N=2768$ observations.

## Abbreviations

ADO: Age at Diabetes Onset
BMI: Body Mass Index
HBA1C: Glycated hemoglobin test
FPG: Fasting Plasma Glucose
RPG: Random Plasma Glucose
HDLC: High-Density Lipoprotein Cholesterol
OGTT: Oral Glucose Tolerance Test
HOMA: Homeostasis Model Assessment
METS-VF: Metabolic Score for Visceral Fat
METS-IR: Metabolic Score for Insulin Resistance
T2DM: Type 2 Diabetes Mellitus
MARD: Mild Age-Related Diabetes
MORD: Mild Obesity-Related Diabetes
SIDD: Severe Insulin-Deficient Diabetes
SIRD: Severe Insulin-Resistant Diabetes
NHANES: National Health and Nutrition Examination Survey
ENSANUT: Encuesta Nacional de Salud y Nutrición
IDF: International Diabetes Federation
SIL: Silhouette index
DB: Davies-Bouldin index
CH: Calinski-Harabasz index
ARI: Adjusted Rand Index
AMI: Adjusted Mutual Information index
FM: Fowlkes-Mallows index
ACC: Accuracy
PRE: Precision
REC: Recall
F1: F1-Score
ROC: Receiver Operating Characteristics
AUC: Area Under the Curve

## Footnotes

1 *National Health and Nutrition Survey*, for english.
