# Supplementary material for: Machine learning based study for the classification of Type 2 diabetes mellitus subtypes
Source: BioData Min. 2023 Aug 22;16:24. doi: 10.1186/s13040-023-00340-2 (PMC10463725; doi:10.1186/s13040-023-00340-2)
Supplement: Supplementary file 1 — Additional file 1. [file 13040_2023_340_MOESM1_ESM.pdf]

Supplementary Material: Machine Learning Based Study for the Classification of Type 2 Diabetes Mellitus Subtypes

Nelson E. Ordoñez-Guillen[1], Jose Luis Gonzalez-Compean[1], Ivan Lopez-Arevalo[1], Miguel Contreras-Murillo[1], and Edwin Aldana-Bobadilla[1]

CINVESTAV Tamaulipas, Carretera Victoria-Soto la Marina km 5.5, Victoria, 87130, Tamaulipas, Mexico

1 Supplementary Figures and Tables

Table S1: Per-class classification results for the different schemes and algorithms. Models trained using bootstrap validation on *Dset A*. Class 0: MARD, Class 1: MORD, Class 2: SIDD, Class 3: SIRD.

Algorithm/Scheme	Class	F1-score	Specificity	Sensitivity	Support
SVM / S1	0	0.9988	1	0.9976	417
	1	0.9944	0.9975	1	178
	2	0.9975	1	0.9951	204
	3	1	1	1	195
K-NN / S1	0	0.9772	0.9696	0.9954	431
	1	0.9387	0.9833	0.9514	185
	2	0.9569	0.9902	0.9524	210
	3	0.9333	0.9964	0.8883	197
MLP / S1	0	0.9988	1	0.9977	427
	1	0.9947	0.9988	0.9947	189
	2	0.9952	0.9988	0.9952	210
	3	0.9926	0.9976	0.9950	202
SNNN / S1	0	0.9801	0.9951	0.9681	407
	1	0.9633	0.9903	0.9657	204
	2	0.9745	0.9926	0.9767	215
	3	0.9606	0.9855	0.9799	199
SVM / S2	0	0.8884	0.8979	0.9078	434
	1	0.8792	0.9790	0.8507	201
	2	0.9265	0.9839	0.9175	206
	3	0.7412	0.9489	0.7368	171
K-NN / S2	0	0.8755	0.8552	0.9273	440
	1	0.8815	0.9726	0.8889	180

Continued on next page...

Table S1 Continued:

	2	0.9153	0.9864	0.8873	213
	3	0.6687	0.9592	0.5936	187
MLP / S2	0	0.8551	0.9027	0.8500	420
	1	0.8962	0.9760	0.9011	182
	2	0.9353	0.9784	0.9435	230
	3	0.6812	0.9303	0.6793	184
SNNN / S2	0	0.8743	0.9154	0.8635	447
	1	0.8715	0.9749	0.8522	203
	2	0.9025	0.9579	0.9614	207
	3	0.6986	0.9417	0.6851	181
SVM / S3	0	0.8304	0.8486	0.8654	416
	1	0.8861	0.9684	0.9021	194
	2	0.8499	0.9682	0.8350	200
	3	0.6807	0.9469	0.6232	207
K-NN / S3	0	0.8513	0.8548	0.8876	445
	1	0.8458	0.9624	0.8500	200
	2	0.8492	0.9548	0.8714	210
	3	0.6334	0.9567	0.5510	196
MLP / S3	0	0.8395	0.8763	0.8423	444
	1	0.8346	0.9542	0.8689	183
	2	0.8090	0.9545	0.8090	199
	3	0.6683	0.9310	0.6394	208
SNNN / S3	0	0.8535	0.8797	0.8708	418
	1	0.8647	0.9579	0.8905	201
	2	0.8551	0.9625	0.8551	207
	3	0.6687	0.9504	0.6154	182
SVM / S4	0	0.9884	0.9880	0.9930	428
	1	0.9668	0.9902	0.9742	194
	2	0.9840	0.9964	0.9840	187
	3	0.9620	0.9951	0.9453	201
K-NN / S4	0	0.9467	0.9313	0.9793	435
	1	0.9082	0.9826	0.8900	200
	2	0.9471	0.9975	0.9091	187
	3	0.8845	0.9793	0.8674	181
MLP / S4	0	0.9930	0.9935	0.9953	428
	1	0.9891	1	0.9785	186
	2	0.9858	0.9964	0.9858	212
	3	0.9907	0.9964	0.9953	213
SNNN / S4	0	0.9422	0.9713	0.9274	413
	1	0.9431	0.9824	0.9522	209
	2	0.9828	0.9925	0.9950	201

Continued on next page...

Table S1 Continued:

	3	0.8641	0.9672	0.8736	182
SVM / S5	0	0.8608	0.8815	0.8833	420
	1	0.9055	0.9697	0.9333	195
	2	0.9299	0.9814	0.9299	214
	3	0.6936	0.9566	0.6316	190
K-NN / S5	0	0.8648	0.8650	0.8956	450
	1	0.8728	0.9689	0.8750	200
	2	0.9120	0.9776	0.9243	185
	3	0.6630	0.9497	0.6000	200
MLP / S5	0	0.8537	0.9085	0.8405	420
	1	0.8942	0.9726	0.8942	208
	2	0.9212	0.9778	0.9303	201
	3	0.6846	0.9240	0.7017	181
SNNN / S5	0	0.8588	0.9066	0.8478	427
	1	0.8895	0.9729	0.8918	194
	2	0.9333	0.9699	0.9760	208
	3	0.6647	0.9361	0.6477	176
SVM / S6	0	0.8587	0.8380	0.9190	420
	1	0.8794	0.9800	0.8497	193
	2	0.9138	0.9731	0.9245	212
	3	0.5923	0.9600	0.5030	169
K-NN / S6	0	0.8366	0.8094	0.8953	449
	1	0.8537	0.9542	0.8883	197
	2	0.9049	0.9760	0.9119	193
	3	0.4658	0.9559	0.3636	187
MLP / S6	0	0.8733	0.8906	0.8918	425
	1	0.8946	0.9779	0.8832	197
	2	0.9216	0.9765	0.9353	201
	3	0.7042	0.9478	0.6684	187
SNNN / S6	0	0.8433	0.8345	0.8967	426
	1	0.8550	0.9573	0.8788	198
	2	0.8986	0.9617	0.9375	208
	3	0.5190	0.9663	0.4032	186
SVM / S7	0	0.8405	0.8137	0.8984	443
	1	0.8421	0.9548	0.8660	194
	2	0.8906	0.9732	0.8953	191
	3	0.4558	0.9481	0.3641	184
K-NN / S7	0	0.8356	0.7847	0.9301	429
	1	0.8471	0.9520	0.8738	206
	2	0.9016	0.9844	0.8777	188
	3	0.3973	0.9538	0.2959	196

Continued on next page...

Table S1 Continued:

MLP / S7	0	0.8341	0.8204	0.8884	430
	1	0.8282	0.9360	0.8934	197
	2	0.8645	0.9728	0.8450	200
	3	0.3916	0.9420	0.3077	182
SNNN / S7	0	0.8389	0.8190	0.9062	405
	1	0.8668	0.9377	0.9458	203
	2	0.9290	0.9811	0.9392	181
	3	0.3636	0.9607	0.2595	185

Table S2: Per-class classification results for the different schemes and algorithms. Models trained using bootstrap validation on *Dset B*. Class 0: MARD, Class 1: MORD, Class 2: SIDD, Class 3: SIRD.

Algorithm/Scheme	Class	F1-score	Specificity	Sensitivity	Support
SVM / S1	0	0.9924	0.9965	0.9892	462
	1	0.9873	0.9937	0.9957	235
	2	0.9885	0.9988	0.9829	175
	3	0.9968	0.9989	1	154
K-NN / S1	0	0.9626	0.9391	1	437
	1	0.9161	0.9908	0.8707	232
	2	0.9438	0.9964	0.9096	166
	3	0.9408	0.9880	0.9438	160
MLP / S1	0	0.9965	0.9947	1	425
	1	0.9956	0.9987	0.9956	225
	2	0.9879	0.9988	0.9819	166
	3	0.9912	0.9988	0.9883	171
SNNN / S1	0	0.9174	0.9007	0.9467	469
	1	0.6193	0.9512	0.5369	203
	2	0.8455	0.9706	0.8382	173
	3	0.9138	0.9645	0.9831	178
SVM / S2	0	0.9952	0.9965	0.9952	413
	1	0.9891	0.9987	0.9826	230
	2	0.9945	0.9975	1	182
	3	0.9970	0.9988	1	165
K-NN / S2	0	0.9866	0.9819	0.9955	443
	1	0.9716	0.9924	0.9716	211
	2	0.9575	0.9939	0.9441	179
	3	0.9907	1	0.9816	163
MLP / S2	0	0.9989	0.9982	1	450
	1	0.9953	0.9987	0.9953	212

Continued on next page...

Table S2 Continued:

	2	0.9969	1	0.9939	164
	3	1	1	1	183
SNNN / S2	0	0.9109	0.8521	0.9833	478
	1	0.6231	0.9507	0.5299	234
	2	0.8653	0.9803	0.8343	181
	3	0.8882	0.9765	0.9085	153
SVM / S3	0	0.9610	0.9604	0.9744	430
	1	0.9331	0.9752	0.9426	244
	2	0.9191	0.9845	0.9138	174
	3	0.7717	0.9670	0.7362	163
K-NN / S3	0	0.9548	0.9627	0.9569	441
	1	0.9025	0.9670	0.9213	216
	2	0.9155	0.9817	0.9130	184
	3	0.7595	0.9608	0.7362	163
MLP / S3	0	0.9622	0.9586	0.9738	458
	1	0.9172	0.9681	0.9391	230
	2	0.9174	0.9856	0.9045	178
	3	0.7653	0.9723	0.7211	147
SNNN / S3	0	0.8798	0.8269	0.9506	466
	1	0.5495	0.9588	0.4329	231
	2	0.7740	0.9288	0.8514	175
	3	0.7727	0.9667	0.7438	160
SVM / S4	0	0.9934	0.9930	0.9956	455
	1	0.9849	0.9962	0.9828	232
	2	0.9973	1	0.9946	185
	3	0.9872	0.9977	0.9872	156
K-NN / S4	0	0.9594	0.9506	0.9820	445
	1	0.9142	0.9836	0.8875	240
	2	0.9314	0.9929	0.9006	181
	3	0.9318	0.9838	0.9458	166
MLP / S4	0	0.9978	0.9965	1	459
	1	0.9912	0.9987	0.9869	229
	2	0.9943	0.9988	0.9943	175
	3	0.9875	0.9977	0.9875	160
SNNN / S4	0	0.9215	0.8816	0.9724	471
	1	0.5424	0.9585	0.4267	225
	2	0.8113	0.9440	0.8834	163
	3	0.8546	0.9627	0.8944	161
SVM / S5	0	0.9966	0.9965	0.9977	441
	1	0.9828	0.9936	0.9871	232
	2	0.9807	0.9976	0.9727	183

Continued on next page...

Table S2 Continued:

	3	0.9877	0.9977	0.9877	163
K-NN / S5	0	0.9593	0.9395	0.9868	454
	1	0.9268	0.9832	0.9179	207
	2	0.9184	0.9926	0.8786	173
	3	0.9072	0.9880	0.8859	149
MLP / S5	0	0.9976	0.9982	0.9976	425
	1	0.9935	0.9987	0.9913	230
	2	0.9972	1	0.9943	176
	3	0.9936	0.9976	1	156
SNNN / S5	0	0.9013	0.8357	0.9954	436
	1	0.4860	0.9706	0.3545	220
	2	0.8270	0.9515	0.8644	177
	3	0.8971	0.9712	0.9290	169
SVM / S6	0	0.9963	0.9965	0.9975	406
	1	0.9913	0.9987	0.9870	231
	2	1	1	1	186
	3	0.9845	0.9964	0.9876	161
K-NN / S6	0	0.9836	0.9784	0.9934	452
	1	0.9689	0.9885	0.9776	223
	2	0.9632	0.9964	0.9458	166
	3	0.9725	0.9988	0.9521	167
MLP / S6	0	0.9937	0.9966	0.9925	398
	1	0.9924	0.9986	0.9887	265
	2	0.9914	0.9975	0.9942	173
	3	0.9935	0.9976	1	153
SNNN / S6	0	0.9847	0.9812	0.9934	454
	1	0.9631	0.9938	0.9487	234
	2	0.9828	0.9954	0.9884	173
	3	0.9803	0.9977	0.9721	179
SVM / S7	0	0.9920	0.9947	0.9909	438
	1	0.9811	0.9922	0.9873	236
	2	0.9851	0.9988	0.9763	169
	3	0.9909	0.9976	0.9939	165
K-NN / S7	0	0.9786	0.9756	0.9849	465
	1	0.9415	0.9874	0.9324	207
	2	0.9432	0.9866	0.9486	175
	3	0.9664	0.9965	0.9536	151
MLP / S7	0	0.9942	0.9982	0.9907	429
	1	0.9819	0.9948	0.9819	221
	2	1	1	1	177
	3	0.9910	0.9964	1	166

Continued on next page...

Table S2 Continued:

SNNN / S7	0	0.9066	0.9171	0.9204	427
	1	0.6937	0.9388	0.6524	210
	2	0.8796	0.9790	0.8579	183
	3	0.9214	0.9695	0.9770	174

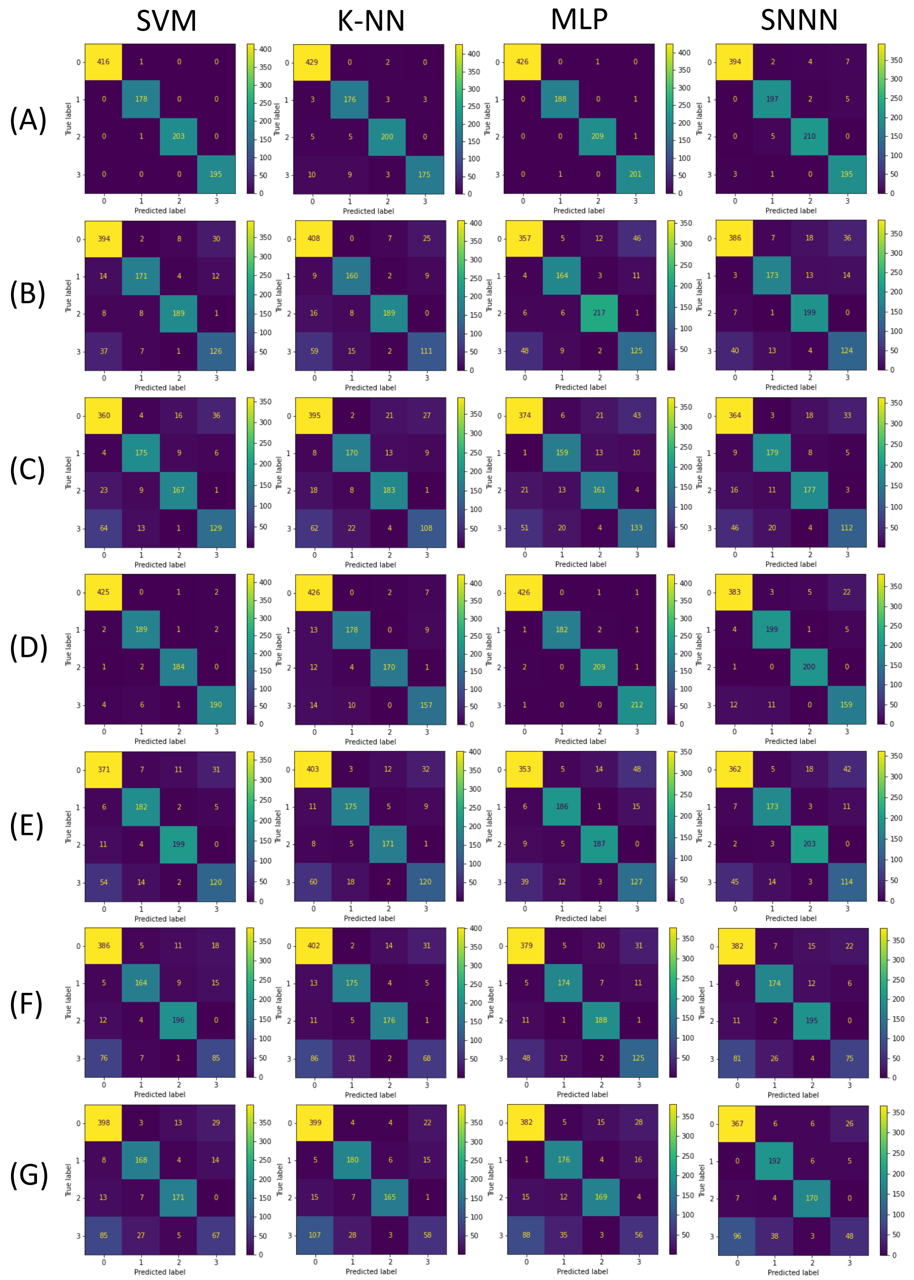

Fig. S1. Confusion matrices of models A trained using bootstrap. Results correspond to best case scenario for all models. Lines (A) to (G) correspond to schemes S1 to S7. Class 0: MARD, Class 1: MORD, Class 2: SIDD, Class 3: SIRD.

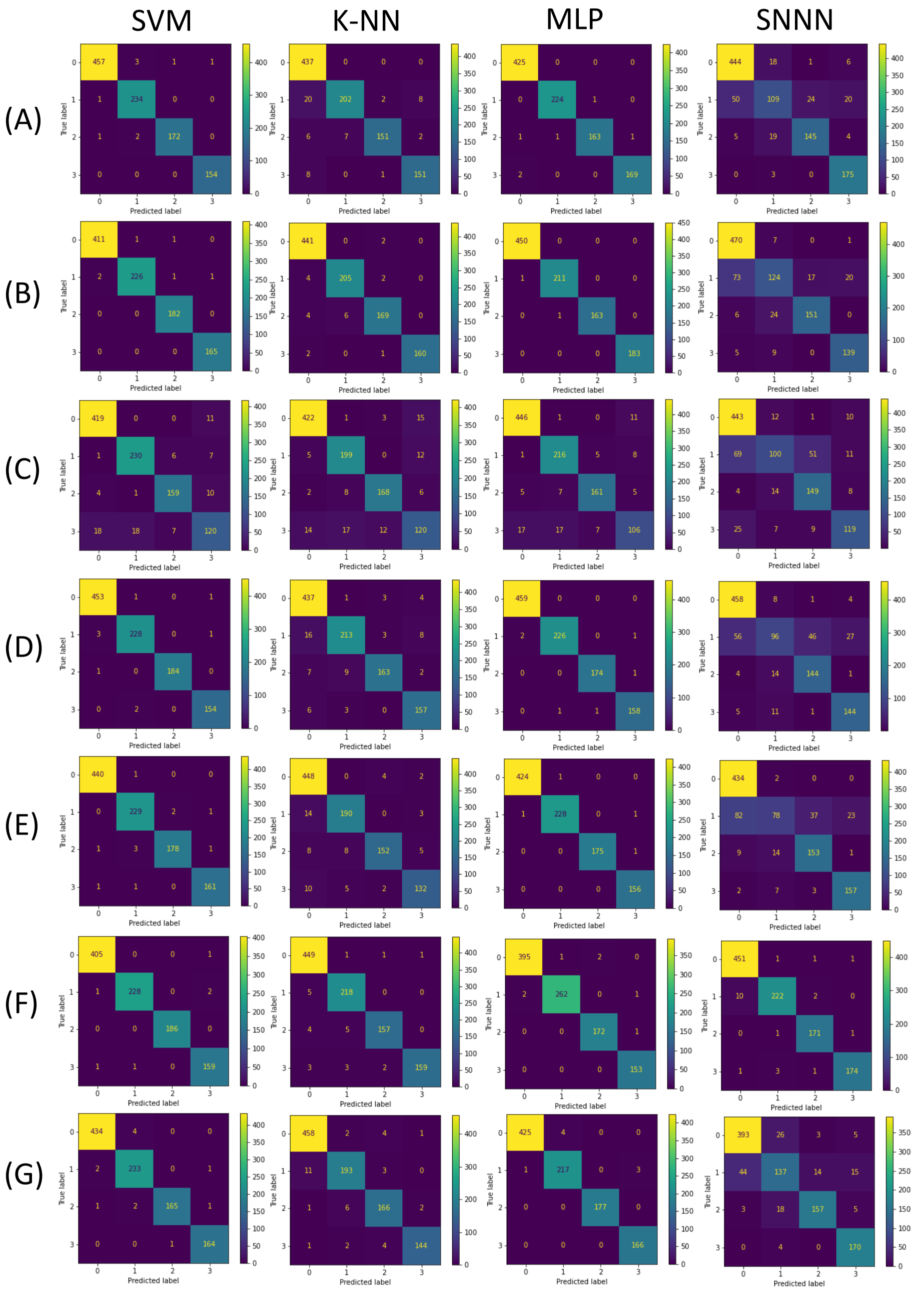

Fig. S2. Confusion matrices of models B trained using bootstrap. Results correspond to best case scenario for all models. Lines (A) to (G) correspond to schemes S1 to S7. Class 0: MARD, Class 1: MORD, Class 2: SIDD, Class 3: SIRD.

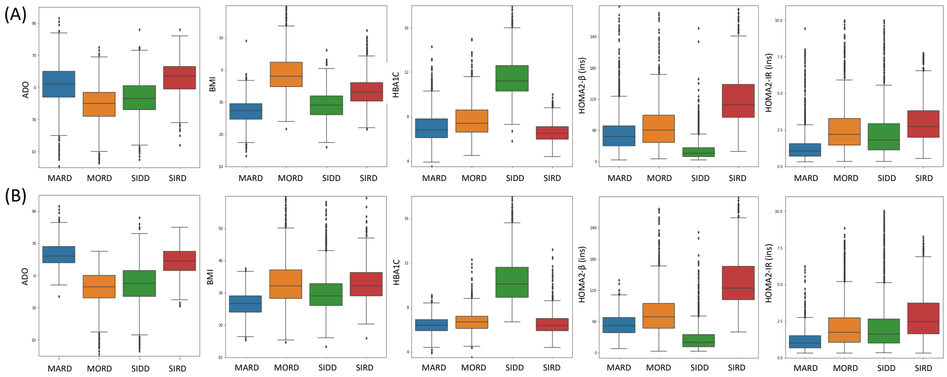

Fig. S3. Distribution patterns of the five involved variables per class. (A) Results obtained from our classification scheme. (B) Results obtained from the classification web tool developed in Bello-Chavolla *et al.*
